# Infrared Photodissociation Spectroscopy of Benzene–V^+^(CO)_n_ “Piano Stool” Cations

**DOI:** 10.1021/acs.jpca.6c03262

**Published:** 2026-07-01

**Authors:** Zachary D. Reed, Michael K. Desouza, Richard B. Odonkor, Michael A. Duncan

**Affiliations:** † Department of Chemistry, 1355 University of Georgia, Athens, Georgia 30602, United States; ‡ Optical Measurements Group, 10833 National Institute of Standards and Technology, 100 Bureau Drive, Gaithersburg, Maryland 20899, United States

## Abstract

Benzene–vanadium–carbonyl cations of the form bz-V^+^(CO)_*n*_ are produced in a pulsed nozzle laser vaporization source and studied by infrared photodissociation spectroscopy and density functional theory. Photofragmentation patterns indicate a stable tricarbonyl core ion. However, infrared spectroscopy and comparison to the predictions of density functional theory computations indicate that the coordination behavior is more nuanced. The small clusters contain a 16-electron tricarbonyl core ion, consistent with the fragmentation patterns. However, larger clusters with more CO ligands begin to form the tetracarbonyl ion with 18 valence electrons. The tricarbonyl and tetracarbonyl species have distinctly different infrared spectra. The asymmetric carbonyl stretch for both complexes is red-shifted from that of gas phase vanadium carbonyl, but the red shift is greater for the tetracarbonyl species.

## Graphical abstract

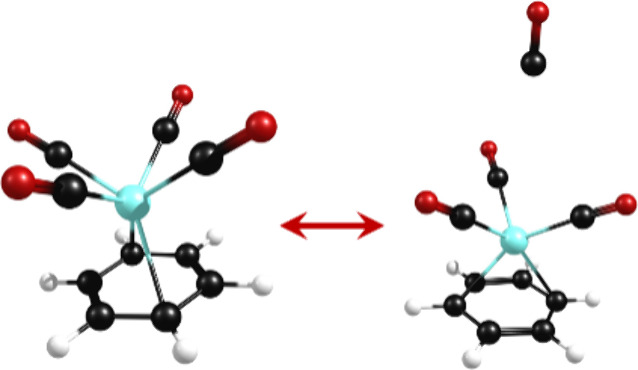

## Introduction

Organometallic carbonyl molecules have been a topic of interest since the discovery of ferrocene and related complexes more than 50 years ago.^1–5^ These complexes have “piano stool” structures, with a planar aromatic molecule (e.g., cyclopentadiene or benzene; indicated hereafter as cp or bz) acting as the “seat” and carbonyls as “legs.” Fischer introduced cp-V­(CO)_4_ and cp-Mn­(CO)_3_ in 1954.^6,7^ Several benzene–metal–carbonyls have also been reported, such as bz-Cr­(CO)_3_, which was synthesized by Fischer in 1957.^8^ Calderazzo synthesized a variety of vanadium–carbonyl–arene complexes, including the bz-V^+^(CO)_4_ cation stabilized with various counterions.^9,10^ It has been shown that the formation of η^6^ or η^5^ complexes between arenes and metal carbonyls significantly alters the reactivity of the arene.^1–5^ For example, the complexation with Cr­(CO)_3_ activates benzene to nucleophilic attack due to electron withdrawing effects from the metal carbonyl.^3^ Such charge transfer effects are typically reflected in the carbonyl stretch vibrations. In the present report, we investigate the details of coordination and charge-transfer in gas phase benzene–vanadium–carbonyl cations using infrared photodissociation spectroscopy of these carbonyl stretch vibrations.

The bz-V^+^(CO)_*n*_ cations are particularly interesting because of the possible coordination numbers that might be formed. The mixed–ligand complexes cp-V­(CO)_4_, cp-Mn­(CO)_3_, and bz-Cr­(CO)_3_ are all stable according to the 18-electron rule.^1^ However, V­(CO)_6_ is stable with 17 electrons.^1^ On the other hand, V^+^(CO)_7_ has 18 electrons, and calculations predicted that this cation should be stable.^11^ Collision induced dissociation measurements found some evidence for the formation of V^+^(CO)_7_.^12^ However, we have investigated the infrared spectroscopy of gas phase vanadium group carbonyl cations, and found no evidence for the formation of a seven-coordinate complex.^13,14^ Instead, we found a stable V^+^(CO)_6_ ion with a symmetric octahedral structure. In that same study, the isoelectronic Nb^+^ ion formed both six- and seven-coordinate carbonyls and Ta^+^ formed exclusively the seven-coordinate species.^13^ According to this previous work, gas phase bz-V^+^(CO)_*n*_ could form either a tricarbonyl, analogous to V^+^(CO)_6_ or the tetracarbonyl species reported previously by Calderazzo.^9,10^

Gas phase infrared photodissociation spectroscopy provides a sensitive probe of the coordination number and the bonding in metal ion–ligand complexes. Our group and others have previously applied this technique to various metal–carbonyl^13–29^ and metal–benzene^30–34^ systems. In particular, we have studied the vanadium carbonyl cations and their oxides,^14,21^ as well as the vanadium–benzene sandwich and half-sandwich cations.^30,31^ Here we apply this technique to mixed benzene–vanadium–carbonyl cations to explore the details of their bonding. The infrared spectra are compared to the predictions of density functional theory computations to further elucidate the structure and bonding of these ions. Previous experiments on vanadium carbonyl ions have shown their unanticipated coordination behavior.^14^ Experiments on vanadium–benzene ions have explored their bonding energetics and the unusual propensity for the formation of sandwiches and multidecker sandwiches.^30,31,35–42^ Both theory and experiments on vanadium–benzene complexes have demonstrated the difficulties in determining the electronic spin states for the vanadium ion.^30,31,41,42^ Vanadium ions also exhibit unusual reactivity with acetylene, producing the V^+^(benzene) ion via a cycloaddition reaction.^43^

## Experimental Section

Benzene–vanadium–carbonyl ionic complexes are produced in a pulsed nozzle laser vaporization source^44^ using the third harmonic of a pulsed Nd:YAG laser (355 nm; Continuum Surelite), which is focused on a rotating and translating 1/4 in. diameter vanadium rod. The rod is mounted with a 3/4 in. horizontal offset in front of a pulsed nozzle (General Valve Series 9). The details of this so-called “offset” source^44^ and the general apparatus^45^ have been described in detail elsewhere. The expansion gas is pure carbon monoxide seeded with benzene, which is introduced via a reservoir placed in the gas line. The nozzle backing pressure was 300 psi. The expansion was skimmed and ions in the resulting molecular beam were pulse-extracted into a home-built reflectron time-of-flight mass spectrometer designed for photodissociation experiments.^46,47^ Ions of the desired composition were mass selected with a pulsed deflection plate and excited with tunable infrared radiation in the turning region of the reflectron. The infrared is generated by an optical parametric oscillator/amplifier (OPO/OPA; LaserVision) laser pumped by the fundamental of a Nd:YAG laser (Continuum 8010). The tuning range is 2000–4500 cm^–1^, the line width is 2 cm^–1^, the absolute energy calibration is ± 2 cm^–1^, and the pulse energy is 1–2 mJ/pulse in the carbonyl stretching region. Complexes with sufficiently weakly bound ligands dissociate upon resonant excitation with infrared light, and spectra are generated by recording the fragment ion yield as a function of the laser frequency.

Density functional theory calculations were performed to investigate the structures, spin configurations, and vibrational spectra of the complexes studied. Singlet, triplet and quintet spin states were examined for each bz-V^+^(CO)_*n*_ complex studied. All calculations were performed in the Gaussian16 program package^48^ using the B3LYP functional. The def2-TZVP basis set was used on all atoms.^49^ A scaling factor of 0.968 was applied to account for anharmonicity of the C–O stretch vibrations.^24^

## Results and Discussion

A mass spectrum of the bz-V^+^(CO)_*n*_ complexes produced is shown in Figure 1. The main series of peaks corresponds to V^+^(CO)_*n*_ masses. The bz-V^+^(CO)_*n*_^+^ ions form a less intense secondary series, which can be seen on the expanded view in the inset. Some water-containing ions are also present, as we find experimentally that water enhances the production of the desired complexes. Water acts as an electron scavenger and inhibits ion–electron recombination in the plasma, thus promoting the formation of cations.^44^ The other small secondary series of mass peaks is therefore H_2_O–V^+^(CO)_*n*_. We produce bz-V^+^(CO)_*n*_ complexes containing up to 10 carbonyls. Mass selection of the bz-V^+^(CO)_*n*_ complexes excludes the unwanted V^+^(CO)_*n*_ and (H_2_O)­V^+^(CO)_*n*_ ions. No bz-V^+^(CO)_*n*_ complex has any noticeably enhanced intensity compared to other ions, and we must investigate the photofragmentation patterns to gain clues about the coordination of these complexes.

**Figure 1:**
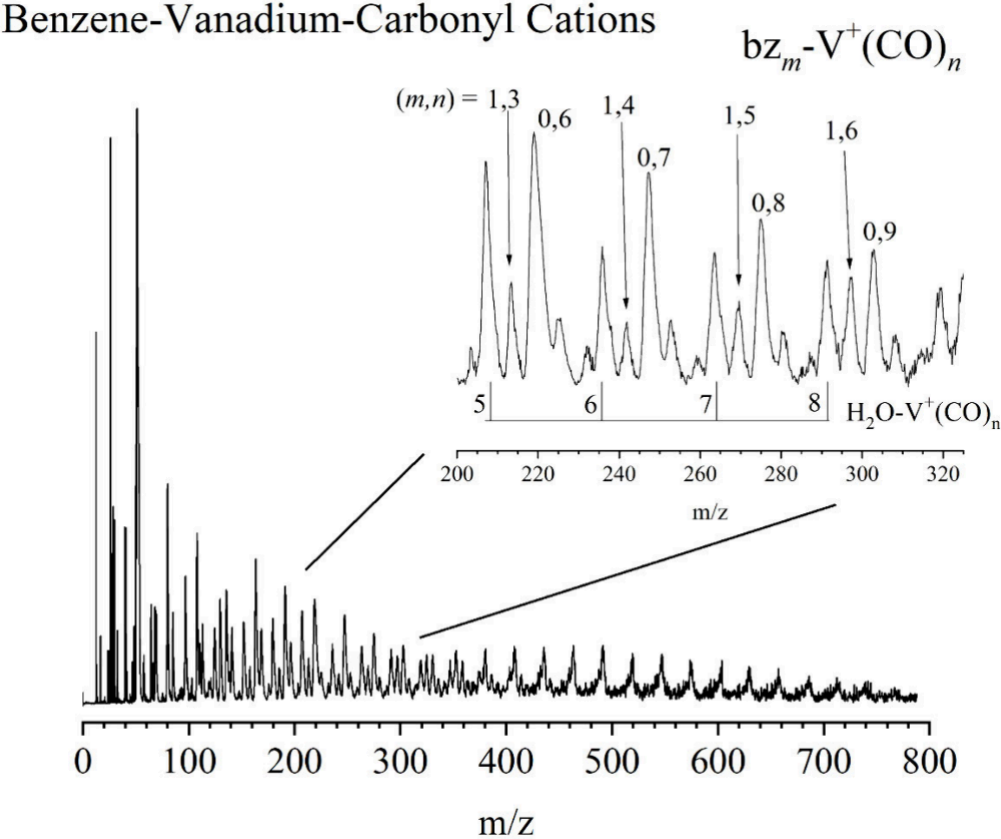
Mass spectrum of bz-V^+^(CO)_*n*_ clusters produced by our cluster source. Inset, expanded view of the mass spectrum with several series labeled. The vertical intensities are in arbitrary units here and in other figures.

The photofragmentation mass spectra of the bz-V^+^(CO)_*n*_ complexes with infrared excitation are presented in Figure 2. These are shown as the difference of the mass spectra with the fragmentation laser “on” minus the laser “off.” The result is a negative-going parent ion mass peak, showing its depletion, and positive fragments. The laser wavelength is selected to maximize the fragmentation of each complex. We do not observe any fragmentation for the bz-V^+^(CO)_1–3_ complexes in the C–O stretching region. The *n* = 4 complex fragments readily on excitation with 2070 cm^–1^ light, and loses a single carbonyl. Larger complexes lose multiple carbonyls and have several fragment ion channels. The *n* = 5 complex loses one or two carbonyls to produce *n* = 3 and *n* = 4. The masses corresponding to the loss of two carbonyls are more intense. The *n* = 6 and 7 complexes lose two carbonyls as their primary decay channel, but the loss of one or three carbonyls is also detected. Taken all together, these results suggest that the *n* = 3 complex survives preferentially, indicating that it has a completed coordination sphere, and that additional carbonyls are bound externally.

**Figure 2:**
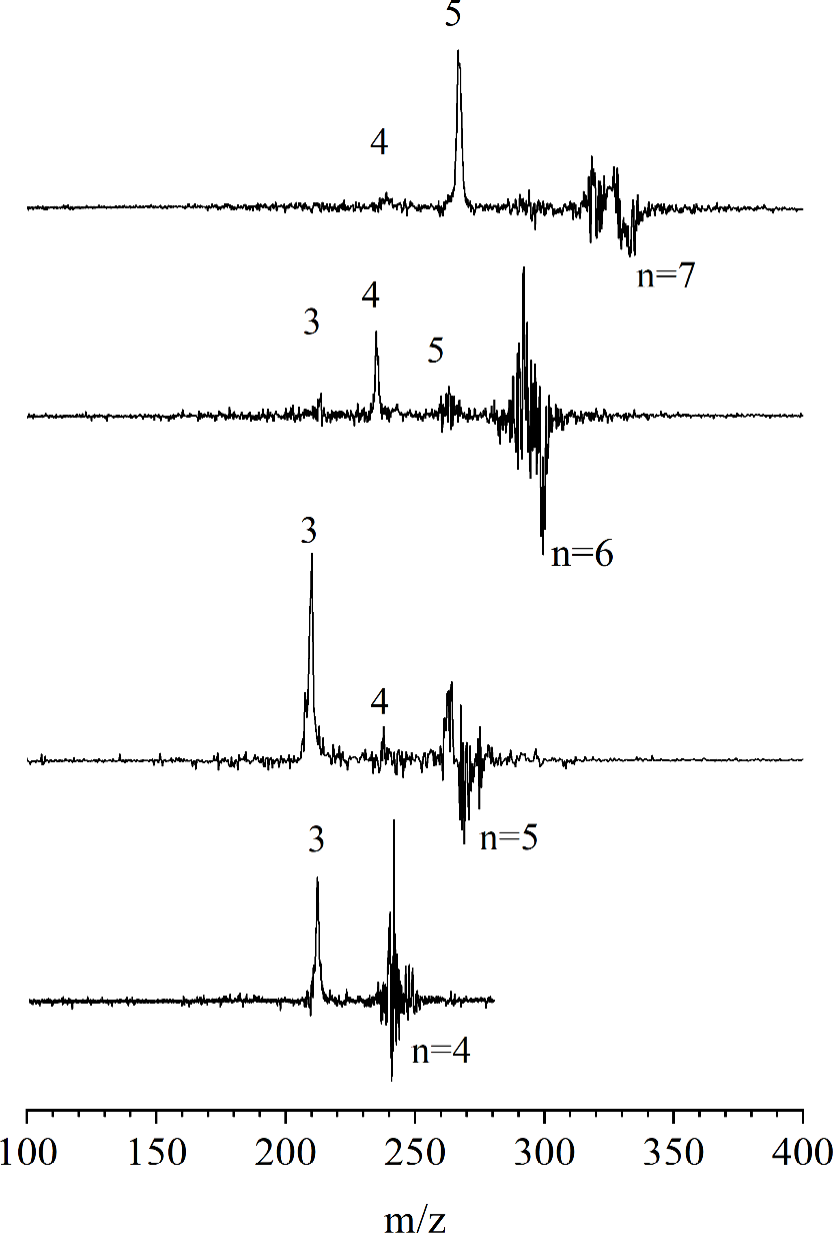
Photofragmentation mass spectra of bz-V^+^(CO)_*n*_, *n* = 4–7. Each of these was collected with the laser on the most intense resonance for the respective cluster size.

The vibrational spectra of these complexes are measured by recording the fragment ion yield versus the frequency in the region of the C–O stretch (2143 cm^–1^ for the isolated CO molecule).^50^ The spectra measured for the *n* = 4–7 complexes are shown in Figure 3. The *n* = 4 spectrum here is measured by the loss of one carbonyl, and those for all the larger complexes are measured by the loss of two carbonyls. The *n* = 4 and 5 complexes have three vibrational bands: two intense ones near 2070 and 2120 cm^–1^, and a weaker one at 2164 cm^–1^. The larger clusters have these same features and additional weaker bands at about 2030 and 2090 cm^–1^, and eventually one for *n* = 7 at 2150 cm^–1^. The dashed red line shows the frequency of gas phase CO at 2143 cm^–1^.^50^ The main bands measured here all fall at frequencies lower than this. The 2150 cm^–1^ band is essentially at the same frequency as gas phase CO, and the 2164 cm^–1^ band is the only one appearing at higher frequency.

**Figure 3:**
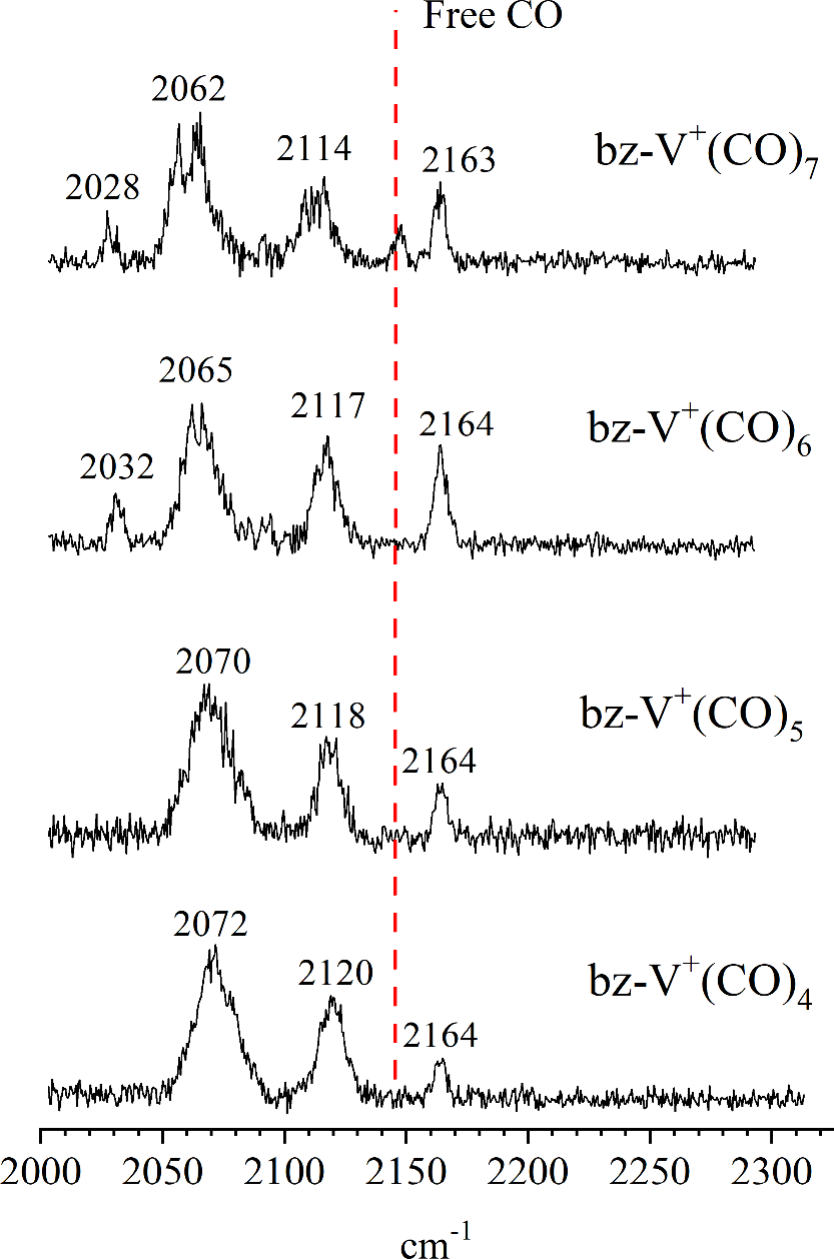
Infrared photodissociation spectra of bz-V^+^(CO)_*n*_, *n* = 4–7. The *n* = 4 complex was measured in the mass channel corresponding to the elimination of one CO, whereas all other cluster sizes were measured in the mass channel corresponding to the loss of two CO’s.

These vibrations can be compared to that of the gas phase V­(CO)_6_^+^ ion, whose single carbonyl stretch band was measured at 2097 cm^–1^.^14^ The two main bands in each of these spectra straddle that frequency, indicating that the cation–carbonyl interactions here are very similar to those in the V^+^(CO)_6_ complex. The bonding in several gas phase metal carbonyl ion systems has been described in previous work.^1,13–29,51–57^ The red shift observed for υ_CO_ in most carbonyl systems is attributed to the dominance of π back-bonding over σ-bonding. π back-bonding leads to increased electron density in the antibonding orbitals of CO, which weakens the bonds and thus causes red-shifted carbonyl vibrations. In this system, we see a red shift comparable to that in the V­(CO)_6_^+^ ion. This suggests that having a benzene ligand opposite the carbonyls has roughly the same effect as having carbonyls in that position. The vanadium cation-benzene binding energy has been previously measured to be 55.8 kcal/mol.^35^ This is much greater than the energy of a single carbonyl bond (roughly 20 kcal/mol), but comparable to the bonding energy of three carbonyls.^12^ If the charge-transfer is related to ligand bonding strength, it is then perhaps understandable that the carbonyl vibrations here are not so different from those in the V­(CO)_6_^+^ ion.

Another aspect of the previous work on V­(CO)_*n*_^+^ ions was that complexes with *n* > 6 were found to have an additional band at 2165 cm^–1^ assigned to externally bound carbonyls. The same resonance was seen for several other metal–carbonyl ions having external ligands.^13–29^ This resonance occurs at slightly higher frequency than the isolated CO value because these external ligands are partially polarized by their attachment to the ion complex. This same external-CO band is detected here for the *n* = 4 complex and all larger ones. The *n* = 3 complex cannot be fragmented, suggesting that it has only more strongly bonded ligands. It therefore has no resonances at all that can be detected with photodissociation, including none at the position for external CO ligands. These combined observations suggest that the *n* = 3 complex represents a filled coordination sphere, consistent with the results of the fragmentation mass spectra. The *n* = 6 and 7 complexes have the same three bands as the *n* = 4 and 5 complexes, with the 2165 cm^–1^ feature growing in intensity. It makes sense that this band gains intensity as more external CO’s are added. We also see two weaker bands emerging near 2030 and 2090 cm^–1^, indicating that some other isomer or spin state may be present.

To explore these structural issues further, we performed DFT computations for the singlet, triplet, and quintet configurations of the (bz)­V^+^(CO)_*n*_ ions for *n* = 1–7. The quintet spin state, which is also the spin of the isolated V^+^ ion, is found to be most stable for the *n* = 1–2 complexes. However, at *n* = 3 the triplet becomes more stable. The *n* = 4 and larger complexes have isomers corresponding to either a stable three-coordinate (3C) or four-coordinate (4C) core ion, with the remaining ligands bound externally. The positions of external ligands are not unique, and so we show representative structures for these isomers. The 3C ions are more stable in the triplet spin state, and the 4C ions are more stable in the singlet state. For convenience, we use the notation (*n*, *m*) to refer to *n* carbonyls directly bound to the metal and *m* externally bound carbonyls. In this system, the bz-V^+^(CO)_4_ complex with one externally bound carbonyl is (3, 1) and the bz-V^+^(CO)_4_ complex with all ligands coordinated directly to the metal is (4, 0). The energetics for all calculated complexes are presented in Table 1. The full details of these computations are provided in the Supporting Information.

**Table 1:** Energetics of bz-V^+^(CO)_*n*_ Complexes^a^

*n*	2*s* + 1	isomer	*E* (hartree)	Rel *E* (kcal/mol)	BDE(CO) (kcal/mol)
0	1		–1175.96	15.4	
0	3		–1175.98	5.3	
0	5		–1175.99	0	
1	1	(1, 0)	–1289.36	14.9	28.9
1	3	(1, 0)	–1289.38	6.6	27
1	5	(1, 0)	–1289.39	0	28.3
2	1	(2, 0)	–1402.77	12.2	28.7
2	3	(2, 0)	–1402.78	4.3	28.2
2	5	(2, 0)	–1402.79	0	26
3	1	(3, 0)	–1516.17	6.4	26.6
3	3	(3, 0)	–1516.18	0	25.2
3	5	(3, 0)	–1516.15	19.7	1.2
4	1	(4, 0)	–1629.56	0	22
4	3	(4, 0)	–1629.47	57.4	–41.8
4	5	(4, 0)	–1629.52	23.6	11.7
4	1	(3, 1)	–1629.53	20.7	1.3
4	3	(3, 1)	–1629.54	14.6	1
4	5	(3, 1)	–1629.51	34.4	0.9
5	1	(4, 1)	–1742.92	0	0.8
5	3	(4, 1)	–1742.89	16.6	41.6
5	5	(4, 1)	–1742.88	23.5	1
5	1	(3, 2)	–1742.89	20.4	2.5
5	3	(3, 2)	–1742.9	14	2.4
5	5	(3, 2)	–1742.86	34.1	2

a Bond dissociation energy (BDE) values are relative to the corresponding bz-V^+^(CO)_*n*−1_ complex with the same spin. Isomers indicated as (*n*, *m*) refer to *n* ligands coordinated to metal and *m* external ligands. The *n* = 4 and 5 complex energies are relative to the lowest energy structures with those ligand numbers.

The electronic spin state is an important consideration in the stable structures. As noted, the (3, 0) complex is most stable as a triplet state. When an additional CO is placed near the metal in the (3, 0) complex, the singlet spin state converges to form the (4, 0) complex. The triplet spin state, however, converges to the (3, 1) complex, with the external carbonyl binding to the first carbonyl coordination sphere. The (3, 1) triplet is 14.6 kcal/mol higher in energy than the (4, 0) singlet. The *n* = 5 complex produces a (3, 2) structure in the triplet state and a (4, 1) structure in the singlet state. A similar trend applies for *n* = 6.

We have calculated the binding energy for carbonyl eliminations from these ions, and the results are also presented in Table 1. These computations employ the B3LYP functional, and thermochemistry like this for transition metal complexes may vary significantly with the functional employed. However, we have investigated several different functionals in recent work from our lab and have found that B3LYP provides the most reliable results, even though other functionals have been recommended for transition metal systems.^58–60^ The first carbonyl attached to the metal ion is bound by 28.3 kcal/mol, the second by 26.0 kcal/mol, and the third by 25.2 kcal/mol. The fourth carbonyl in the singlet (4, 0) complex is bound by 22.0 kcal/mol. We would not expect to observe infrared fragmentation for these complexes, because the photon energies are around 2000 cm^–1^ and these binding energies are roughly 8000–10,000 cm^–1^. Multiphoton dissociation would be required, and our per-pulse energies are not high enough for this. Armentrout has previously measured the binding energy for the homoleptic V­(CO)_*n*_^+^ system, and found the binding energy to be around 20 kcal/mol for *n* = 1–7, with some variation for each complex.^12^ Our calculated binding energy here for the singlet (4, 0) complex is consistent with those measurements, and indicates that the carbonyl is directly coordinated to the metal ion. However, the computed binding energy for the fourth carbonyl in the (3, 1) structure is only 1.0 kcal/mol (350 cm^–1^). This CO can be eliminated easily with the infrared photons in the carbonyl stretching region. Similar low binding energies are found for all of the larger complexes with externally bound ligands. As shown, the *n* = 5 and 6 complexes lose fewer ligands than should be possible on the basis of simple energy accounting. This is likely because of energy dissipation throughout the cluster by intramolecular vibrational redistribution (IVR), or to a finite rate of dissociation slower than the instrument time scale of about 1 μs.

The spectra predicted for the different *n* = 4 isomers are compared to our experimental spectrum in Figure 4. The geometries of each complex are also shown there. Each has two main bands and a smaller band corresponding to the external CO stretch. We see reasonably good agreement between the predicted (3, 1) triplet band positions and our experimental data. The predicted doublet at 2053/2058 cm^–1^ coincides with the broadened 2072 cm^–1^ experimental band. The spectrum predicted for the (3, 1) quintet also agrees fairly well with the experimental spectrum (see Supporting Information), and there are no significant spectral differences to rule out one or the other, although the quintet is computed to be substantially higher in energy. The spectrum predicted for the (4, 0) complex does not match well with our experiment, and we would not expect to be able to fragment this complex anyway because of its high computed binding energy (see Table 1). Its two bands are predicted to be at lower frequencies than those in the experiment, and it should not have any band near 2164 cm^–1^ from external CO ligands. The calculated spectrum for the (3, 1) singlet (not shown) has band positions similar to those of the (4, 0) complex. Therefore, the *n* = 4 complex detected here is likely a triplet with a V­(CO)_3_^+^ core and an externally bound carbonyl. Because the (4, 0) complex has a higher dissociation energy, it might be present in the beam, but could not be detected in this photodissociation experiment on the *n* = 4 complex.

**Figure 4:**
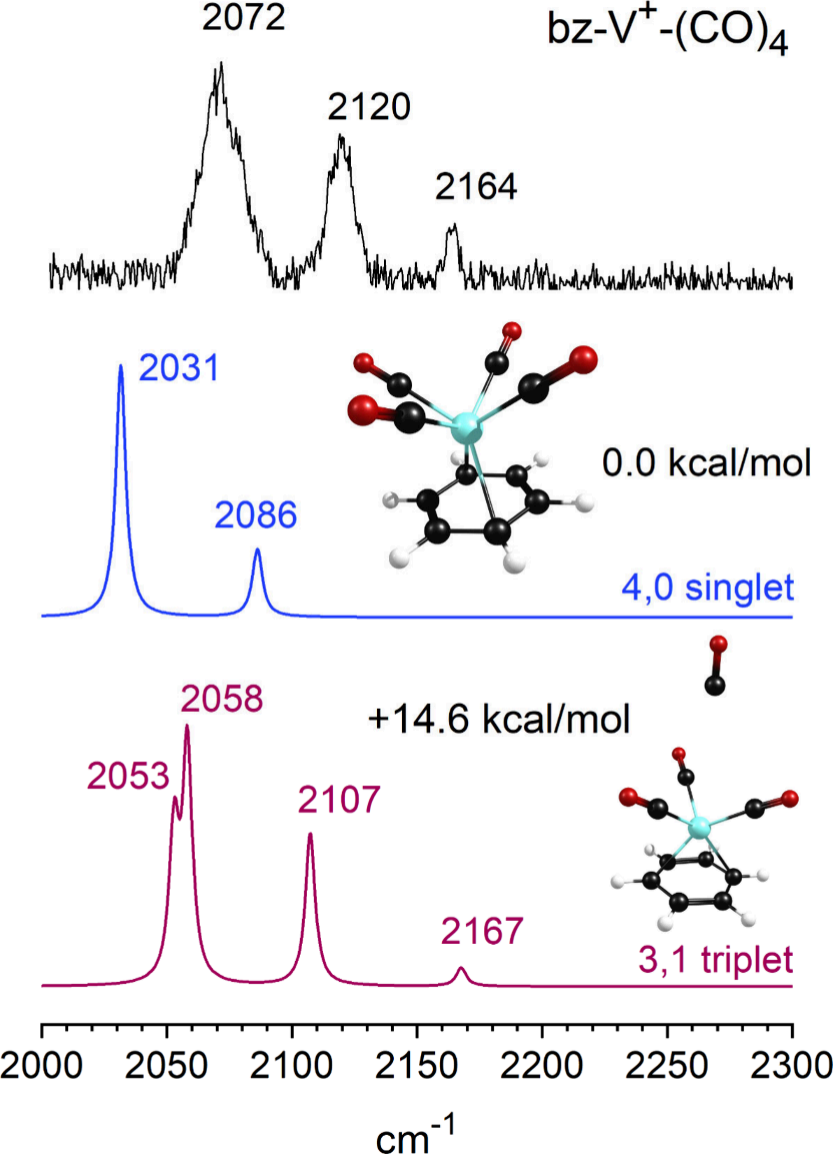
Infrared spectra of bz-V^+^(CO)_4_ compared to the spectra predicted by theory, along with calculated structures. Complexes are indicated as (*n*, *m*) with *n* directly coordinated carbonyls and *m* external carbonyls.

In the (3, 0) and (3, 1) triplets, the 2070 cm^–1^ band corresponds to two asymmetric carbonyl stretches, split by some 5 cm^–1^. The 2120 cm^–1^ band is a symmetrical carbonyl stretch. These two bands are respectively red-shifted and blue-shifted relative to the V­(CO)_6_^+^ gas phase frequency, which has a single vibrational band at 2097 cm^–1^, assigned to the nearly degenerate asymmetric CO stretches in that complex.^14^ The (4, 0) singlet band at 2031 cm^–1^ is a doubly degenerate asymmetrical carbonyl vibration, and the 2086 cm^–1^ band is a symmetric carbonyl stretch. This fully symmetric C–O stretching vibration has low oscillator strengths (generally <1 km/mol) in our previous metal carbonyl cation studies, but here the benzene breaks the symmetry and leads to strongly IR-active vibrations, with computed oscillator strengths of >500 km/mol.

To investigate the spectra of larger complexes, we performed calculations on various *n* = 5 isomers. For this complex, we have two different experimental spectra, one measured by the loss of one CO and one measured by the loss of two. The calculated and experimentally measured spectra for *n* = 5, measured in the mass channel corresponding to the loss of two CO’s, is shown in Figure 5. As shown in Figure 3, this spectrum looks nearly identical to that of *n* = 4, measured in the loss-of-one-CO channel. Two isomers are identified by theory for this complex, with (4, 1) and (3, 2) coordination. The spectrum predicted for these two structures both have two main bands and a high frequency band corresponding to one or more external CO ligands. Because both structures have external ligands and their binding energies are low, it is understandable that we observe efficient photodissociation for this cluster. The band positions for the (3, 2) structure match the experimental band positions somewhat better than those of the (4, 1) structure. Additionally, the loss-of-two-CO fragment mass is much more intense than the loss-of-one-CO mass (see Figure 2), which would be expected for the (4, 1) isomer. Finally, the band positions for this ion are virtually the same as those for the *n* = 4 complex, which is concluded to have the (3, 1) structure. For all these reasons, we conclude that the vibrational bands detected here are those of the (3, 2) isomer, even though it is predicted to be less stable than the (4, 1) isomer.

**Figure 5:**
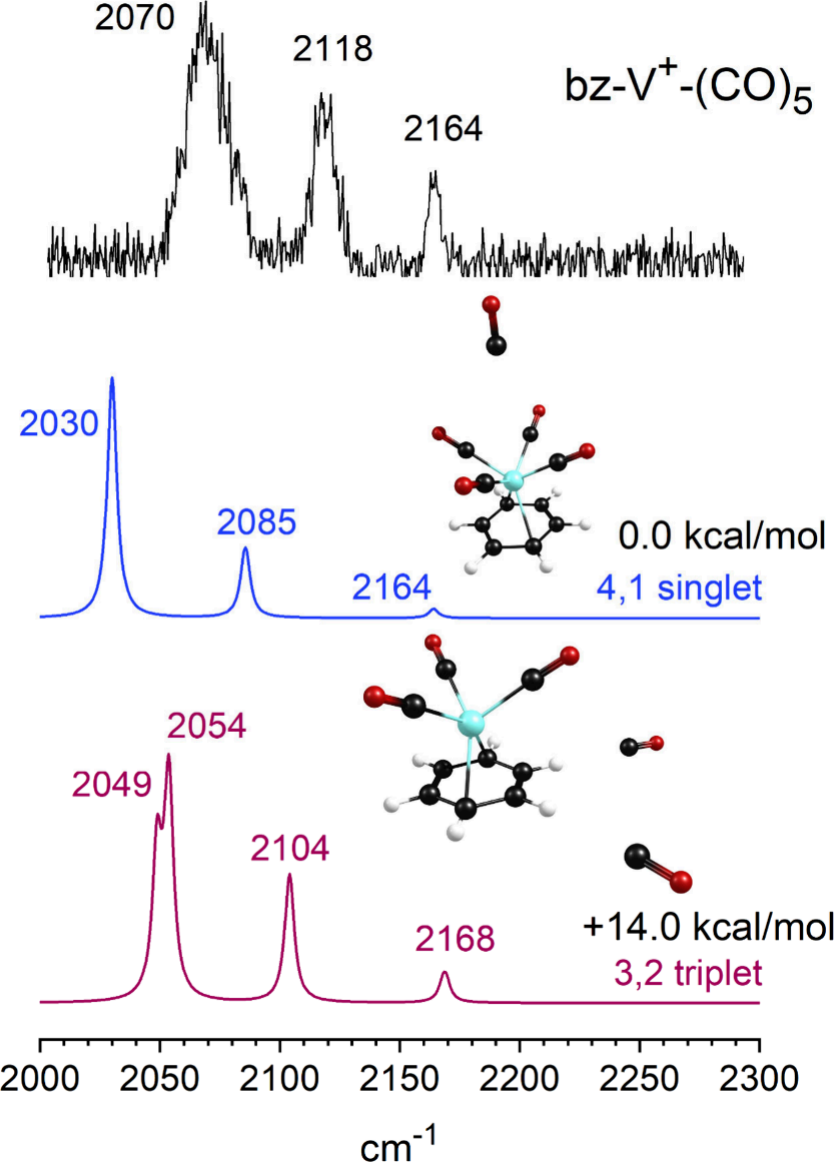
Infrared spectra of bz-V^+^(CO)_5_ measured in the mass channel corresponding to the loss of two CO’s compared to the spectra predicted by theory, along with calculated structures. Complexes are indicated as (*n*, *m*) with *n* directly coordinated carbonyls and *m* external carbonyls.

Figure 6 shows the spectrum of the *n* = 5 complex measured in the mass channel corresponding to the loss of one CO ligand compared to the same two isomer structures considered in Figure 5. Again, there are two main red-shifted bands at 2067 and 2128 cm^–1^ and an intense band at 2164 cm^–1^ corresponding to an external CO vibration. Additionally, there are two new bands at 2032 and 2094 cm^–1^. Figure 3 shows that these new bands persist in the *n* = 6 and 7 cluster sizes. These new bands match nicely with the computed vibrations for the (4, 1) isomer. Apparently, the four-coordinate isomer, which has 18 electrons and was synthesized previously by Calderazzo,^9,10^ is also produced here. It makes sense that it is not detected in the loss-of-two-CO’s mass channel, because only the (3, 2) isomer has two ligands with bonding weak enough to be eliminated by infrared excitation. The loss-of-one-CO mass channel can in principle detect both the (3, 2) and the (4, 1) isomers, and it allows the (4, 1) to be detected. However, the (3, 2) vibrational pattern is still more intense. The IR oscillator strengths of these two isomers are similar, with computed intensities for both near 1000 km/mol. We can therefore conclude that the population of complexes with the three-coordinate core is greater than that with the four-coordinate, even though the four-coordinate species is predicted to be more stable. This same conclusion then also applies for the *n* = 6 and 7 complexes.

**Figure 6:**
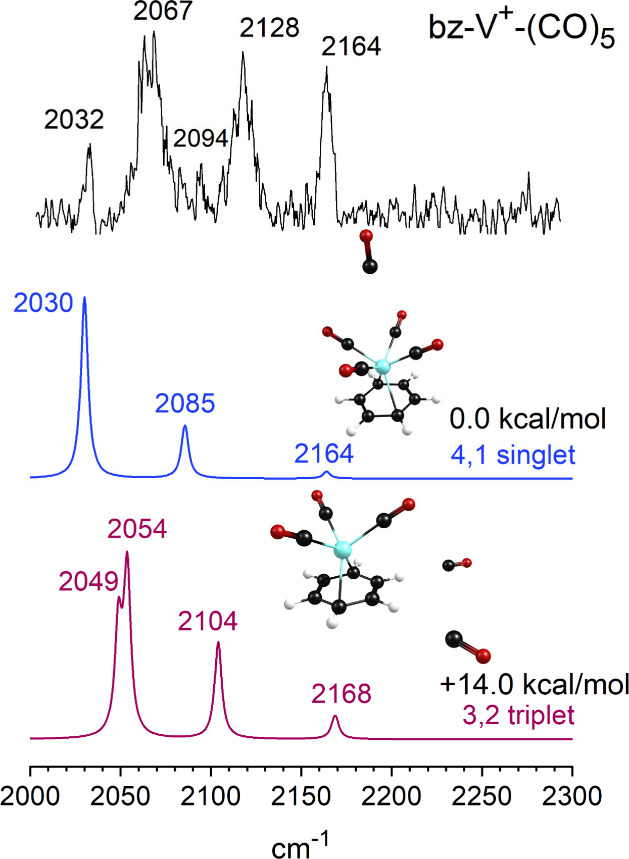
Infrared spectra of bz-V^+^(CO)_5_ measured in the mass channel corresponding to the loss of one CO compared to the spectra predicted by theory, along with calculated structures. Complexes are indicated as (*n*, *m*) with *n* directly coordinated carbonyls and *m* external carbonyls.

The coordination behavior seen here is reminiscent of what we observed in the V^+^(CO)_*n*_ system.^14^ In that system, the seven-coordinate ion V^+^(CO)_7_, which has 18 electrons, was predicted to be most stable, but the V^+^(CO)_6_ 16-electron ion was actually observed. Here, bz-V^+^(CO)_4_ is the 18-electron ion predicted to be most stable, but bz-V^+^(CO)_3_ is formed in greater abundance. In the pure-carbonyl ions, the seven-coordinate 18-electron ion was not detected at all, whereas here the corresponding ion is detected at lower abundance. In the pure carbonyl ions, we also studied Nb^+^ and Ta^+^ complexes,^13^ and both produced the seven-coordinate species. As we discussed in the previous work, there are several factors that might explain these coordination trends. The vanadium cation is smaller than the niobium or tantalum cations, and the accommodation of seven CO ligands around it may be sterically inhibited. In the present bz-V^+^(CO)_*n*_ system, benzene is smaller than three carbonyls,^3^ and the reduced crowding may allow the formation of the 18-electron species. However, the most likely factor in these systems is the kinetics of growth and how this is affected by the metal ion spin state. In both the V^+^(CO)_6_ and bz-V^+^(CO)_3_ ions, the most stable structure has a triplet spin. On the other hand, both the V^+^(CO)_7_ and bz-V^+^(CO)_4_ ions are most stable in the singlet spin state. Adding an extra CO to either V^+^(CO)_6_ or bz-V^+^(CO)_3_ requires a spin change to reach the stable ground state for the respective V^+^(CO)_7_ and bz-V^+^(CO)_4_ ions. This spin change is likely to inhibit the rate of growth of the more stable ions, perhaps by introducing an activation barrier to this reaction coordinate. If there is a barrier, the low temperatures of our experiment (typically 20–50 K) would severely limit the spin-change reaction. As we discussed for the pure carbonyls, the heavier metals in the Nb^+^(CO)_*n*_ and Ta^+^(CO)_*n*_ ions may have promoted the required spin changes through enhanced spin–orbit coupling.^13^ If this is true, the Nb^+^ and Ta^+^ should more easily produce the stable bz-M^+^(CO)_4_ complexes. A final issue worth mentioning is the significant uncertainties in spin state energies for DFT computations. If the relative energies of triplet and singlet states is misjudged by DFT, then this whole discussion is moot. Although functionals other than B3LYP are sometimes recommended for transition metal systems,^58–60^ our experience is that these functionals do not perform as well as B3LYP for bonding energetics.^61,62^ Other research has shown that no DFT functionals are completely reliable for spin state energies.^63^

The vibrations measured here for the bz-V^+^(CO)_4_ complex are shifted further to the red than those of the bz-V^+^(CO)_3_ complex. This is quite surprising, because the π back-bonding interactions that cause the red shift are determined by the same metal ion *d* electron density, but distributed over more ligands. Our initial expectation is that the red shift might be smaller for the complex with more ligands. However, the bonding in these ligands is also stronger, which may be an added consideration in these frequencies. An additional factor may be the increased mass of more ligands in motion, which would favor the lower frequencies.

We also measured the spectroscopy of these complexes in the C–H stretching region. Again, no fragmentation was observed for *n* ≤ 3. All complexes for *n* ≥ 4 fragmented very weakly over a broad region, some 200 cm^–1^ wide and centered around 3100 cm^–1^. Our theoretical calculations predict a number of very weak (oscillator strengths of 1–10 km/mol) bands in this region, which explains the weak fragmentation. High fragmentation laser power (∼10 mJ/pulse) was also required to observe any fragmentation in the C–H stretching region, and this may have caused some power broadening. Another consideration could conceivably be a slow rate of IVR from the benzene C–H stretches to the carbonyl ligands which are eliminated.

Calderazzo synthesized [bz-V­(CO)_4_]^+^ complexed with various anions,^9,10^ and obtained infrared spectra. The band positions reported in that work are compared with our gas phase data in Table 2. The solution data is compared to the bands in the bz-V^+^(CO)_5_ spectrum, measured by the loss of one carbonyl, which are assigned to bz-V^+^(CO)_4_. The solution spectra find bands in the general region of our spectra, however none of those bands match ours. Also, there are more C–O stretches reported in that work than should be possible for the bz-V^+^(CO)_4_ ion. It is likely that the solution environment and the counterions present perturb the spectrum beyond recognition.

**Table 2:** Comparison of Various Measured Vibrational Frequencies for bz-V^+^(CO)_4_ Complexes

Complex	Experimental frequencies (cm^–1^)
bz-V^+^(CO)_4_ ^a^	2032, 2094
[bz-V(CO)_4_][V(CO)_6_]^b^	2068 (w), 2018 (vw), 1986 (m),1895 (wm), 1859 (vs)
[bz-V(CO)_4_][PF_6_]^c^	2070, ∼2017 (vvw), 1988

a This work, bands assigned to bz-V^+^(CO)_4_.

b In tetrahydrofuran, reference 9.

c In acetone, reference 9.

## Conclusions

We have produced bz-V^+^(CO)_*n*_ complexes in the gas phase using laser vaporization and investigated them using infrared photodissociation spectroscopy, complemented by DFT computational chemistry. Although theory predicts that the 18-electron complex bz-V^+^(CO)_4_ is the most stable structure, experiments find more efficient production of the 16-electron species bz-V^+^(CO)_3_. This three-carbonyl complex persists as the main core ion in all the larger bz-V^+^(CO)_*n*_ complexes. Beginning at bz-V^+^(CO)_5_, a small amount of the four-carbonyl complex begins to be observed and this minor concentration is also found in the larger clusters. Apparently, a kinetic barrier, likely associated with the required spin change on the metal, inhibits the formation of the stable 18-electron bz-V^+^(CO)_4_ complexes. This behavior is the same as that seen previously in the V^+^(CO)_*n*_ complexes, where the seven-carbonyl species was predicted to be stable, but the V^+^(CO)_6_ ion was actually formed.

Charge transfer interactions in these bz-V^+^(CO)_*n*_ complexes are similar to those in the corresponding V^+^(CO)_*n*_ ions. The carbonyl stretches are slightly red-shifted from the C–O stretch in the isolated ligand, but are roughly the same with or without the benzene ligand. These carbonyl stretches are easily documented in the gas phase measurements described here, but are completely unrecognizable in previously studied condensed phase samples. Surprisingly, the four-carbonyl complex has its vibrations shifted further to the red from that the isolated CO molecule than those of the three-carbonyl complexes.

## Supplementary Material



## Notes

Full details of the DFT computations done in support of the spectroscopy, including the structures, geometries, energetics and vibrational frequencies for each of the structures considered, IR spectra (PDF)
