# Supplementary material for: Infrared Photodissociation Spectroscopy of Benzene–V+(CO)n “Piano Stool” Cations
Source: J Phys Chem A. 2026 Jul 1;130(28):5527–34. doi: 10.1021/acs.jpca.6c03262 (PMC13383745; doi:10.1021/acs.jpca.6c03262)
Supplement: Supplementary file 1 [file jp6c03262_si_001.pdf]

Supporting Information

Infrared Photodissociation Spectroscopy of Benzene-$V^+(CO)_n$ "Piano Stool" Cations

Zachery D. Reed,[2] Michael K. Desouza,[1] Richard B. Odonkor,[1] Michael A. Duncan[1]*

[1]Department of Chemistry, University of Georgia, Athens, Georgia 30602

[2]Optical Measurements Group, National Institute of Standards and Technology, 100 Bureau Drive, Gaithersburg, Maryland 20899

*Email: maduncan@uga.edu

Table of Contents

Computational details	S3
**(CO)**	
Table S1: energy and Figure S5: structure	S4
Figure S1: structural parameters & unscaled vibrational frequencies	S5
**V^+bz**	
Table S2: relative energies	S6
Figure S2 – S4: structural parameters & unscaled vibrational frequencies	S7 – S9
**Bz-V^+(CO)**	
Table S3: relative energies	S10
Figures S5 – S7 : structural parameters & unscaled vibrational frequencies	S11 – S13
**Bz-$V^+(CO)_2$**	
Table S4: relative energies	S14
Figures S8 – S10 : structural parameters & unscaled vibrational frequencies	S15 – S17
**Bz-$V^+(CO)_3$**	
Table S5: relative energies	S18
Figures S11 – S13: structural parameters & unscaled vibrational frequencies	S19 – S21
**Bz-$V^+(CO)_4$**	
Table S6: relative energies	S22
Figures S14 – S20: structural parameters & unscaled vibrational frequencies	S23 – S28
Figure S21 – S22: simulated spectra	S29 – S30
**Bz-$V^+(CO)_5$**	
Table S8: relative energies	S31
Figures S23 – S28: structural parameters & unscaled vibrational frequencies	S32 – S37
Figures S29 – S30: simulated spectra	S38 – S39
**Bz-$V^+(CO)_6$**	
Table S9: relative energies	S40
Figures S31 – S33: structural parameters & unscaled vibrational frequencies	S41 – S43
**Bz-$V^+(CO)_7$**	
Table S11: relative energies	S44
Figures S34 – S36 : structural parameters & unscaled vibrational frequencies	S45 – S47

Computational details;

All calculations were performed using DFT at the B3LYP/def2-TZVP level. The thresholds for energy and structure optimizations were set to “tight,” and all calculations used the default “ultrafine” integration grid. The structures presented were checked for electronic wavefunction stability with the “stable=opt” keyword. All structures are free of imaginary vibrational frequencies, and all electronic energies are zero-point vibrational energy (ZPVE) corrected. Listed vibrational frequencies (cm-1) are unscaled with intensities (km/mol). A calculation of the vibrational frequency of CO with B3LYP/def2TZVP provided a scaling factor of 0.968, with which all simulated spectra shown are scaled

Table S1. CO calculated at the B3LYP/def2-TZVP level of theory using Gaussian16.

2s + 1	Energy (Hartree)	Relative Energy (kcal/mol)
1	-113.35748	0.0

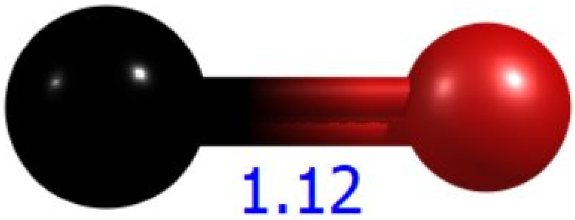

Figure S1. The optimized geometry of CO followed by its predicted frequencies (cm^{-1}) and IR intensities (km/mol).

Frequency (cm^{-1})	Intensity (km/mol)
2214.6097	76.6748

Table S2. V^+bz electronic energy calculated at the B3LYP/def2-TZVP level.

2s + 1	Energy (Hartree)	Relative Energy (kcal/mol)
1	-1175.961346	+15.4
3	-1175.977524	+5.3
5	-1175.985915	0.0

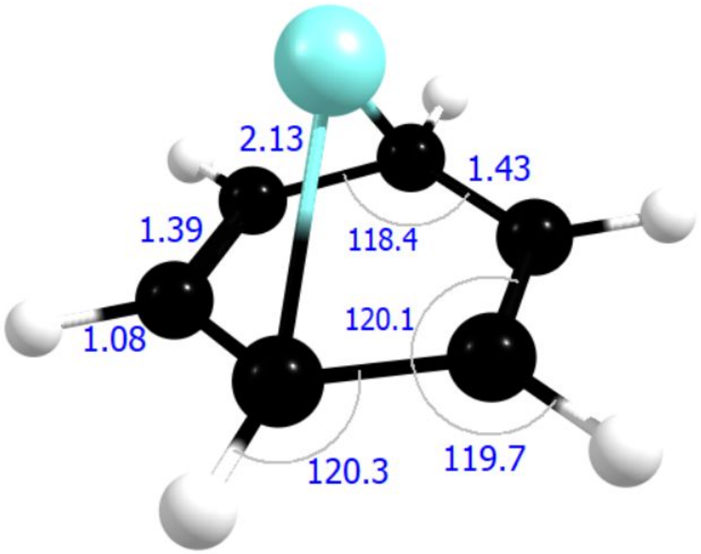

Figure S2. The optimized geometry of singlet V^+bz followed by its predicted frequencies (cm^{-1}) and IR intensities (km/mol).

Frequency (cm^{-1})	Intensity (km/mol)	Frequency (cm^{-1})	Intensity (km/mol)
229.4009	0	1035.7964	0.1259
314.9927	0.2661	1114.0624	0
357.725	0.3844	1169.7055	0.3245
358.5252	1.139	1181.1936	0.103
395.9529	1.442	1331.7715	27.8132
597.3129	0.0855	1360.4297	0
609.1204	0	1437.6243	0
644.6639	5.6735	1456.2859	8.6341
810.426	98.78	1489.7799	2.7043
853.4401	11.3332	1562.8271	6.153
895.0674	1.2423	3189.2346	0.4537
906.2454	4.2755	3190.9314	0
962.4525	0	3198.843	0.0544
971.3533	0.1091	3202.574	5.3369
982.9146	6.7042	3212.0726	8.426
996.9884	6.6814	3214.8241	0.5485
1031.451	0.7469		

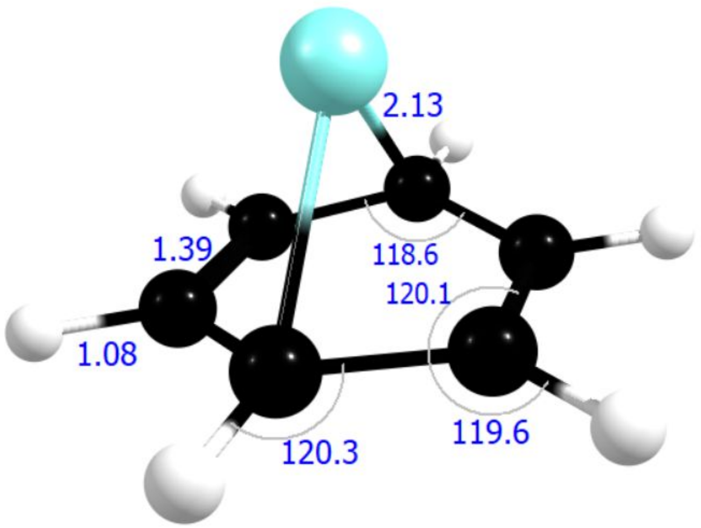

Figure S3. The optimized geometry of triplet V^{+}bz followed by its predicted frequencies (cm^{-1}) and IR intensities (km/mol).

Frequency (cm^{-1})	Intensity (km/mol)	Frequency (cm^{-1})	Intensity (km/mol)
257.6929	0	1034.7913	0.3694
321.7531	0.1717	1107.0335	0
348.0445	0.4112	1165.1007	0.4199
366.3603	0.4789	1179.1448	0.2367
401.5586	0.8069	1327.4569	25.9228
599.4404	0.0349	1357.4189	0
609.5916	0	1427.1058	0
634.3978	5.4414	1452.8099	6.5778
805.3025	99.5113	1484.5914	2.1101
835.4977	11.0562	1552.8971	7.1075
884.6304	1.0665	3191.1599	0.6354
889.2637	5.6815	3193.1295	0
967.8255	0.405	3201.4132	0.0246
969.7038	0	3204.5351	6.7381
975.3018	3.7908	3216.3683	11.31
990.6057	11.5811	3219.0535	0.7548
1028.7774	1.2259		

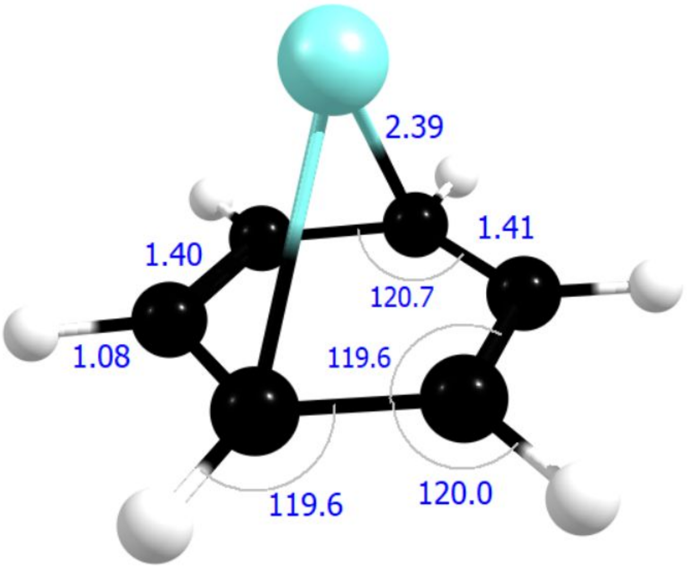

Figure S4. The optimized geometry of quintet V^{+}bz followed by its predicted frequencies (cm^{-1}) and IR intensities (km/mol).

Frequency (cm^{-1})	Intensity (km/mol)	Frequency (cm^{-1})	Intensity (km/mol)
160.2443	6.383	1044.7021	3.3611
218.5021	0.0204	1183.1694	0.0218
219.9283	0	1184.0438	0.1226
242.3198	0.6737	1184.2919	0.0002
414.6673	0.1249	1325.2366	0.0518
547.8121	0	1381.0782	0
615.3611	0.199	1487.9658	16.2482
676.0423	0.0078	1496.5568	11.7791
762.4635	86.6342	1557.4902	0
895.6551	1.7077	1571.6178	0.6604
912.9375	1.5693	3191.5015	0.2241
984.2833	0.4338	3194.9701	0.0571
988.0666	0	3197.8899	0
1000.8566	0.0217	3206.411	5.1555
1008.6152	0.0054	3210.9415	7.1079
1029.8628	4.658	3214.9183	0.1178
1038.0744	0.0052		

Table S3. bz-V^+(CO) electronic energy calculated at the B3LYP/def2-TZVP level.

2s + 1	Energy (Hartree)	Relative Energy (kcal/mol)
1	-1289.364829	+14.9
3	-1289.378086	+6.6
5	-1289.388545	0.0

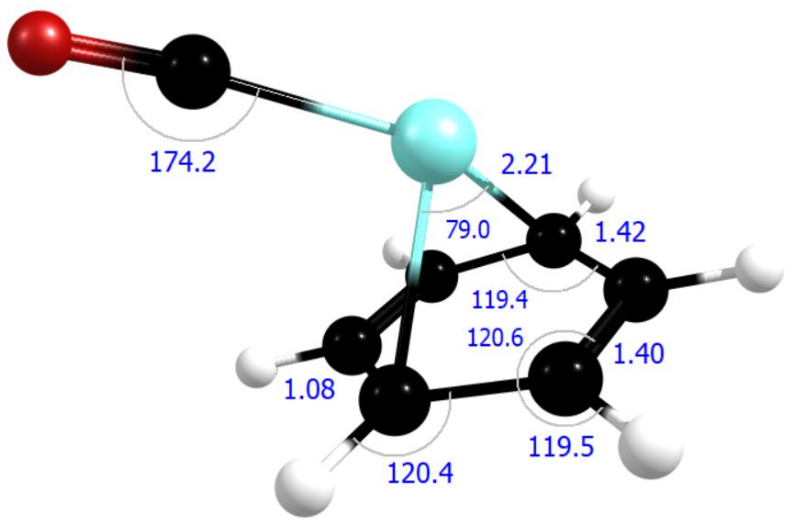

Figure S5. The optimized geometry of singlet bz-V^+(CO) followed by its predicted frequencies (cm^{-1}) and IR intensities (km/mol).

Frequency (cm^{-1})	Intensity (km/mol)	Frequency (cm^{-1})	Intensity (km/mol)
37.802	2.2753	1010.7722	4.812
91.0592	1.2974	1039.866	0.1654
261.65	0.6088	1041.7606	0.6331
280.5465	4.109	1144.1866	1.3949
287.8702	21.6987	1178.9851	1.2165
308.0379	6.7067	1183.9681	4.4355
358.1364	30.0447	1344.7888	16.7985
398.043	9.6356	1370.7988	0.059
417.5706	5.3608	1470.3946	8.4065
437.2855	5.4967	1482.6208	7.5875
599.2408	0.0623	1493.0292	7.3371
615.5176	0.1758	1562.824	28.1484
658.9139	2.1807	2128.5974	1031.4171
798.5272	57.7354	3190.2626	0.5206
884.0844	2.0778	3196.4602	0.1242
908.8308	0.5705	3200.7164	2.1429
937.6952	10.8392	3207.7526	7.9003
978.4962	1.1763	3211.6412	5.1986
986.5715	1.0894	3222.742	3.2977
1001.9274	3.704		

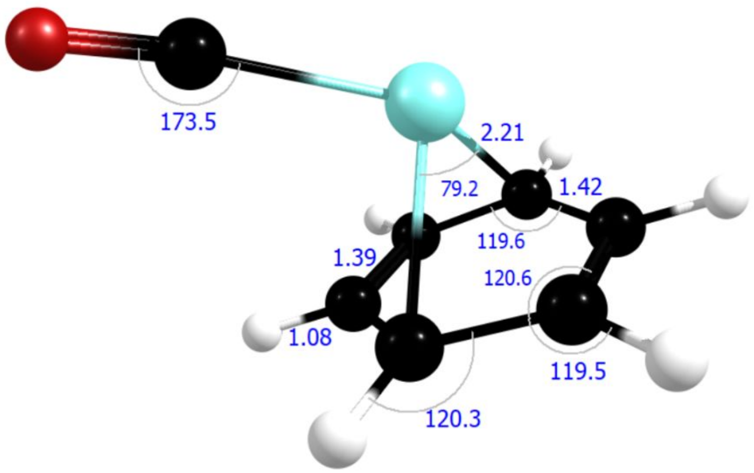

Figure S6. The optimized geometry of triplet bz-V^+(CO) followed by its predicted frequencies (cm^{-1}) and IR intensities (km/mol).

Frequency (cm^{-1})	Intensity (km/mol)	Frequency (cm^{-1})	Intensity (km/mol)
34.2992	2.1738	1007.9443	6.8712
93.8398	1.7677	1039.0721	1.2198
259.4597	1.08	1040.1643	0.095
283.3732	2.6502	1137.6396	1.6747
302.3785	15.2731	1177.0324	0.9658
306.1802	5.3546	1183.3514	5.4915
350.9905	38.5185	1346.9517	18.0016
385.2455	2.0029	1368.3792	0.1615
409.5531	5.7372	1463.7112	3.8134
436.1726	1.8666	1473.4081	11.2473
599.8947	0.2373	1489.5706	5.7975
616.1803	0.1179	1560.0255	31.9171
653.9574	2.0856	2139.1351	1009.4303
794.8788	60.2363	3192.47	0.5526
870.9456	2.0264	3198.7828	0.1404
900.8399	0.7646	3201.5247	2.2815
929.0893	11.5464	3208.6104	9.4495
975.1357	2.1469	3214.3127	6.8258
981.9736	2.1729	3226.5637	3.8467
997.4632	4.6176		

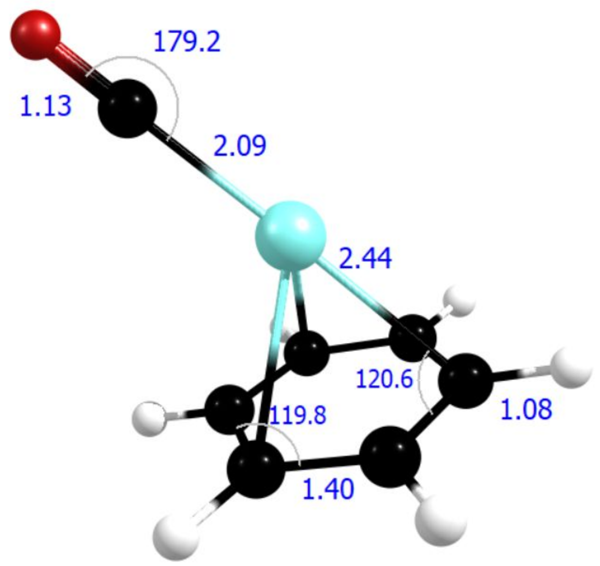

Figure S7. The optimized geometry of quintet bz-V^+(CO) followed by its predicted frequencies (cm^{-1}) and IR intensities (km/mol).

Frequency (cm^{-1})	Intensity (km/mol)	Frequency (cm^{-1})	Intensity (km/mol)
11.2956	2.1707	1036.4656	2.6477
66.9106	0.873	1042.1048	0.0007
205.3199	1.1687	1046.2127	2.8448
212.5443	2.1963	1185.7165	0.3112
226.7992	0.2286	1188.8944	0.098
283.017	0.4827	1190.9728	0.032
336.5371	1.4243	1328.1068	1.514
368.3828	17.7202	1383.2806	0
408.9066	0.585	1493.0285	18.3821
418.6769	0.0937	1499.2206	15.8949
611.081	1.5367	1567.7989	0.0184
615.5635	0.2492	1578.232	0.2852
683.5986	0.097	2151.836	955.1348
765.4159	65.759	3192.5713	0.2912
904.8999	0.9644	3198.0871	0.0387
918.7388	0.3095	3201.4711	0.4967
988.5459	0.5348	3208.1099	6.4336
1001.1113	0.1209	3211.6202	8.9311
1009.2187	0.0763	3215.9024	0.7209
1016.6176	0.2793		

Table S4. bz-$V^+(CO)_2$ electronic energy calculated at the B3LYP/def2-TZVP level.

Isomer	2s+1	Energy (Hartree)	Relative Energy (kcal/mol)
2C	1	-1402.768033	+12.2
2C	3	-1402.780556	+4.3
2C	5	-1402.787441	0.0

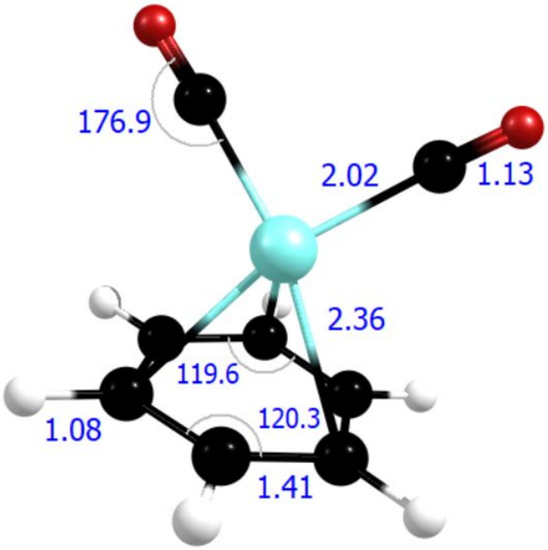

Figure S8. The optimized geometry of isomer 2C-singlet bz-$V^+(CO)_2$ followed by its predicted frequencies (cm^{-1}) and IR intensities (km/mol).

Frequency (cm^{-1})	Intensity (km/mol)	Frequency (cm^{-1})	Intensity (km/mol)
8.7212	0.2882	1002.0385	3.4503
73.5461	1.5476	1014.686	1.4984
84.3172	1.3832	1022.5257	4.4905
88.0479	0.9779	1044.0207	0.5726
247.1104	10.0201	1050.104	0.2446
264.315	4.025	1170.6452	3.296
274.3503	0.9669	1186.7691	1.3402
312.9572	0.3664	1189.1027	5.023
347.3739	38.7999	1350.1826	11.8052
359.7049	1.9716	1378.7638	0.0418
385.1216	3.0791	1484.0108	14.3643
388.2871	15.2921	1497.8794	14.6207
420.5303	2.3847	1523.6671	6.3086
463.0252	23.1256	1573.582	33.9034
514.6353	25.5203	2104.0256	871.7989
606.0403	0.2584	2144.55	883.6901
615.4317	0.443	3192.7786	0.9596
673.4237	3.0553	3200.9661	0.0393
790.0383	46.4917	3201.1156	2.6634
901.8683	0.8208	3211.796	9.2699
916.8083	0.005	3212.5632	5.6057
973.6363	6.1027	3221.0526	3.3699
986.1512	1.6645		

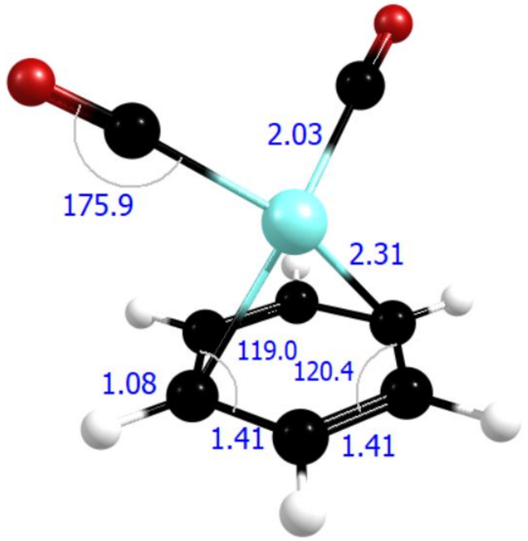

Figure S9. The optimized geometry of isomer 2C-triplet bz-$V^+(CO)_2$ followed by its predicted frequencies (cm^{-1}) and IR intensities (km/mol).

Frequency (cm^{-1})	Intensity (km/mol)	Frequency (cm^{-1})	Intensity (km/mol)
23.0252	0.1102	1009.0387	0.6305
76.3472	0.9991	1016.5418	1.2974
79.7203	1.8049	1024.2296	3.2873
89.0069	0.9537	1040.4945	1.7187
248.2071	12.918	1043.9714	0.0096
250.386	0.2963	1162.6884	1.2888
279.1943	1.2573	1186.2575	0.7309
311.5616	0.0605	1192.2275	6.2879
348.4796	38.72	1358.6641	4.5281
362.4787	1.6021	1377.2929	0.0057
368.9013	3.1784	1481.9538	7.9488
383.1229	10.1967	1492.9527	14.1375
421.6724	0.6718	1525.8964	8.7477
452.2111	22.172	1573.7941	23.5209
517.3156	33.962	2117.3441	847.0825
611.7209	0.4668	2150.6539	858.9478
613.7329	0.2173	3191.6019	0.7483
671.5911	0.0566	3199.457	1.0224
789.0529	47.8005	3201.4812	2.5861
899.622	0.0094	3209.4348	5.2848
917.0181	0.3652	3222.8079	10.5638
971.1923	8.7778	3224.2679	2.3549
983.0657	0.6822		

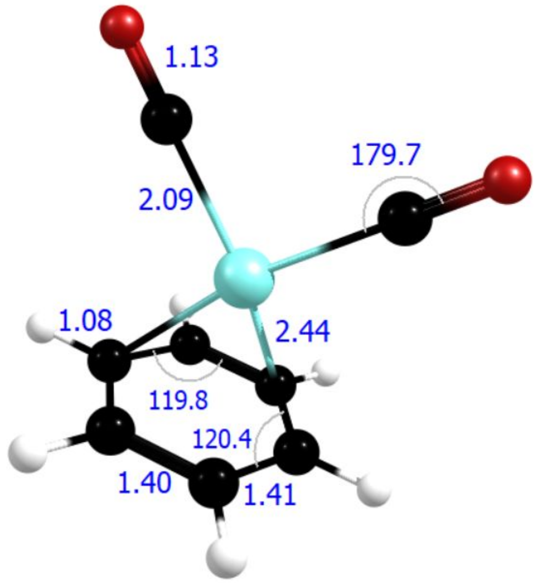

Figure S10. The optimized geometry of isomer 2C-quintet bz-$V^+(CO)_2$ followed by its predicted frequencies (cm^{-1}) and IR intensities (km/mol).

Frequency (cm^{-1})	Intensity (km/mol)	Frequency (cm^{-1})	Intensity (km/mol)
1.7808	0	1015.1204	0.3887
65.8936	0.6441	1021.7478	0.0214
71.2623	0.0042	1040.174	1.0719
74.6448	0.256	1045.2124	0.0721
198.8947	0.3705	1048.8037	1.536
217.678	1.4546	1188.3632	0.0162
222.7816	1.6882	1192.8114	0.0902
282.2575	0	1194.0376	0
314.7199	0.0974	1334.6473	0.0403
345.9853	6.0637	1385.2617	0
359.4594	25.8746	1496.5882	20.4002
362.1428	9.7468	1502.3411	19.5234
410.9477	0.0142	1575.2161	0.0001
411.6688	8.7108	1584.6348	0.0193
420.1768	0.2347	2142.2319	701.2578
615.0154	0	2176.688	743.5024
615.3386	0.1673	3195.2981	0.097
688.511	0.0165	3199.703	0.0242
767.8089	61.3077	3202.452	0
911.9316	0.0341	3209.6862	6.5029
923.075	0.4123	3212.3428	7.6183
992.1916	0.185	3216.721	1.0642
1009.0082	0.0001		

Table S5. bz-$V^+(CO)_3$ electronic energy calculated at the B3LYP/def2-TZVP level.

Isomer	2s+1	Energy (Hartree)	Relative Energy (kcal/mol)
3C	1	-1516.167909	+6.4
3C	3	-1516.178144	0.0
3C	5	-1516.146783	+19.7

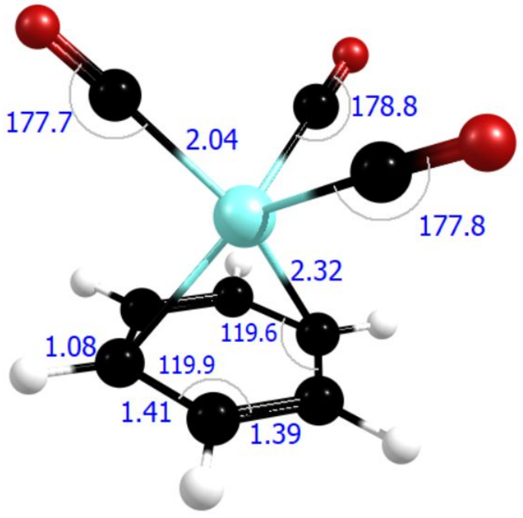

Figure S11. The optimized geometry of singlet bz-$V^+(CO)_3$ followed by its predicted frequencies (cm^{-1}) and IR intensities (km/mol).

Frequency (cm^{-1})	Intensity (km/mol)	Frequency (cm^{-1})	Intensity (km/mol)
8.11	0.0532	979.6821	7.3249
59.4358	0.1828	988.2752	0.8413
71.1026	1.0082	1016.8979	4.1794
77.8835	2.1005	1020.3675	0.2343
81.2719	1.5706	1026.045	1.9811
96.2707	0.2894	1045.9127	0.078
235.5267	2.0391	1051.4786	0.5801
245.7309	2.7519	1180.3787	2.78
261.4467	3.5868	1189.0865	0.866
317.7717	0.3671	1192.0112	3.3586
333.0526	9.0234	1359.4244	11.2895
346.1545	11.2924	1381.1904	0.0091
365.9061	10.6502	1489.1527	12.3786
388.09	2.963	1499.2024	13.7758
394.1222	9.2041	1547.5077	17.4568
415.3358	21.5337	1576.6295	23.2369
427.8897	27.2825	2108.5494	631.8273
432.7684	15.3205	2116.4031	1055.8228
501.3298	35.1174	2166.2166	667.8257
507.6836	21.1655	3197.8469	0.1792
606.6942	0.4475	3201.1126	0.3311
615.6429	0.2362	3205.42	2.4241
677.8368	1.1573	3211.9306	7.9008
791.455	44.7701	3215.752	8.6015
913.4196	0.3297	3220.672	2.7038
923.877	0.5574		

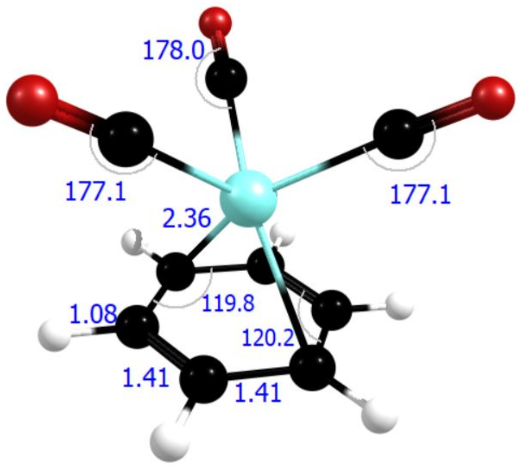

Figure S12. The optimized geometry of triplet bz-$V^{+}(CO)_3$ followed by its predicted frequencies (cm^{-1}) and IR intensities (km/mol).

Frequency (cm^{-1})	Intensity (km/mol)	Frequency (cm^{-1})	Intensity (km/mol)
7.023	0.3064	906.5561	0.1312
46.1976	3.4374	915.6392	0.9595
62.3003	0.0154	964.8848	14.9019
76.7302	1.3891	985.0001	1.2723
80.8734	1.4063	1018.1157	0.0334
96.302	0.4237	1018.6059	4.0136
234.1591	8.4652	1023.4255	3.1664
234.9629	1.8031	1043.9303	0.1884
253.3388	3.3511	1048.1971	1.4235
313.2141	2.3582	1181.3915	4.7722
318.3199	1.637	1182.2481	4.731
336.0835	11.7352	1189.0838	1.3862
360.2971	3.2292	1363.516	7.9629
367.8126	13.8025	1379.0917	0.0084
385.0917	10.2549	1487.5528	11.4893
407.2024	18.5242	1492.9043	7.1784
432.3626	31.0575	1552.5558	23.1088
434.0537	8.2003	1555.9109	42.3146
488.0684	49.8804	2121.0928	652.6555
492.7671	17.6273	2125.7314	1056.4005
607.4111	0.7091	2176.722	629.2757
617.8373	0.0665	3198.4767	0.5755
674.8825	0.9971	3200.8387	0.1616
788.9021	47.9074	3207.6183	3.9446

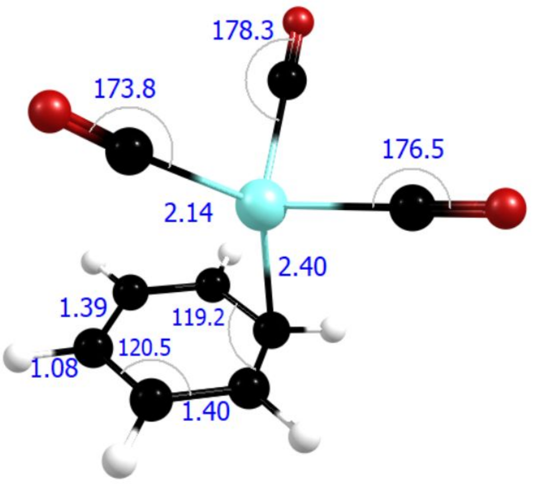

Figure S13. The optimized geometry of quintet bz-$V^+(CO)_3$ followed by its predicted frequencies (cm^{-1}) and IR intensities (km/mol).

Frequency (cm^{-1})	Intensity (km/mol)	Frequency (cm^{-1})	Intensity (km/mol)
18.5219	0.0144	988.9514	0.6429
27.4532	0.0655	1004.1277	4.268
54.9294	0.3154	1015.418	1.0156
59.9374	0.8417	1028.03	0.5769
65.8711	0.6712	1040.4655	2.0342
69.1812	0.1693	1048.5807	1.1973
87.6106	1.286	1054.8197	1.3478
111.5685	0.5221	1184.0812	0.2021
193.0217	3.0036	1195.658	0.3507
258.5959	0.8623	1202.0702	2.0258
266.7131	3.0232	1325.7869	1.8146
286.1198	2.4298	1389.1948	0.0235
298.1695	0.4768	1503.9578	19.2054
306.6253	6.4246	1509.1971	18.7255
315.4633	9.371	1601.1524	5.9685
336.9887	14.3522	1602.4206	6.7943
366.3984	8.2041	2148.7966	915.5319
391.5928	7.9526	2157.8034	514.4079
396.6195	0.7668	2196.3816	446.0066
428.6206	0.1037	3181.8583	0.1845
612.6002	0.0107	3188.6308	0.3515
613.7103	0.2938	3191.6549	1.1865
700.1197	7.8545	3200.5602	1.0206
742.692	87.6649	3201.071	1.4218
896.451	0.153	3210.7593	0.3766
908.436	0.1397		

Table S6. bz-$V^{+}(CO)_4$ electronic energy calculated at the B3LYP/def2-TZVP level.

Isomer	2s+1	Energy (Hartree)	Relative Energy (kcal/mol)
4C	1	-1629.560446	0.0
4C	3	-1629.469028	+57.4
4C	5	-1629.522875	+23.6
3C + 1	1	-1629.527474	+6.1
3C + 1	3	-1629.537198	0.0
3C + 1	5	-1629.505713	+19.8

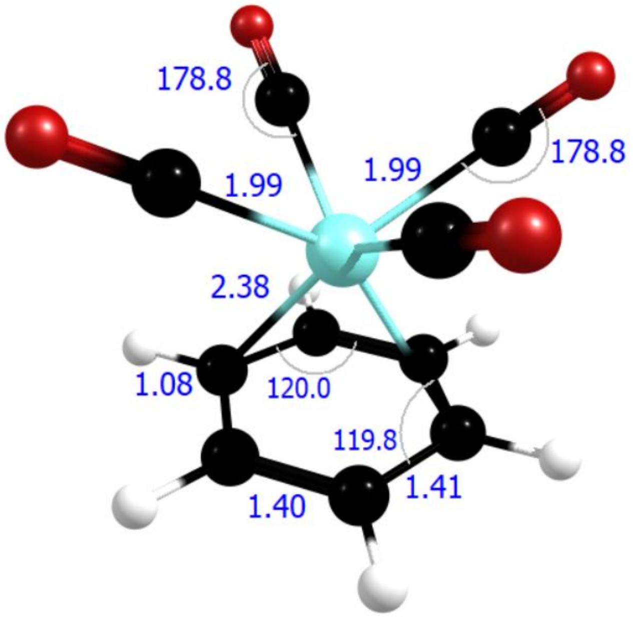

Figure S14. The optimized geometry of isomer 4C-singlet bz-$V^+(CO)_4$ followed by its predicted frequencies (cm^{-1}) and IR intensities (km/mol).

Frequency	Intensity	Frequency	Intensity	Frequency	Intensity
6.0629	0	463.9655	35.8941	1191.6184	0.0238
69.1156	0	467.1133	34.6022	1195.4275	0.2964
94.0736	0.9344	583.2182	52.089	1199.3135	3.9873
94.4788	0.9439	584.9922	53.4136	1369.6734	2.4783
102.3592	3.6124	585.5875	0.0112	1387.1723	0
108.9268	0.0071	610.2371	4.7131	1498.0465	21.9029
118.9179	0.6684	615.5904	10.431	1507.5384	23.6369
120.4741	0.4418	615.9869	35.3776	1569.8921	0.1276
237.1035	0.567	688.3118	0.0253	1595.2903	20.3216
244.8101	2.0592	801.474	41.0772	2098.487	1115.4678
268.7529	0.1844	932.5345	2.4278	2098.9503	1111.067
325.522	0	944.329	2.9086	2110.481	0.0003
367.8659	6.5696	996.7086	0.1829	2155.0927	596.6983
375.6292	0.0065	1010.535	4.4528	3197.8641	0.4469
408.4391	8.4772	1025.2236	0.039	3201.0841	0.0027
409.0213	10.8617	1033.5705	0.0436	3205.4158	0.1976
426.3486	0.6635	1037.5537	0.3338	3211.029	8.3265
436.5311	0	1054.6278	0.084	3215.6256	9.2561
445.9897	0.002	1055.7474	0.0164	3218.854	0.9367

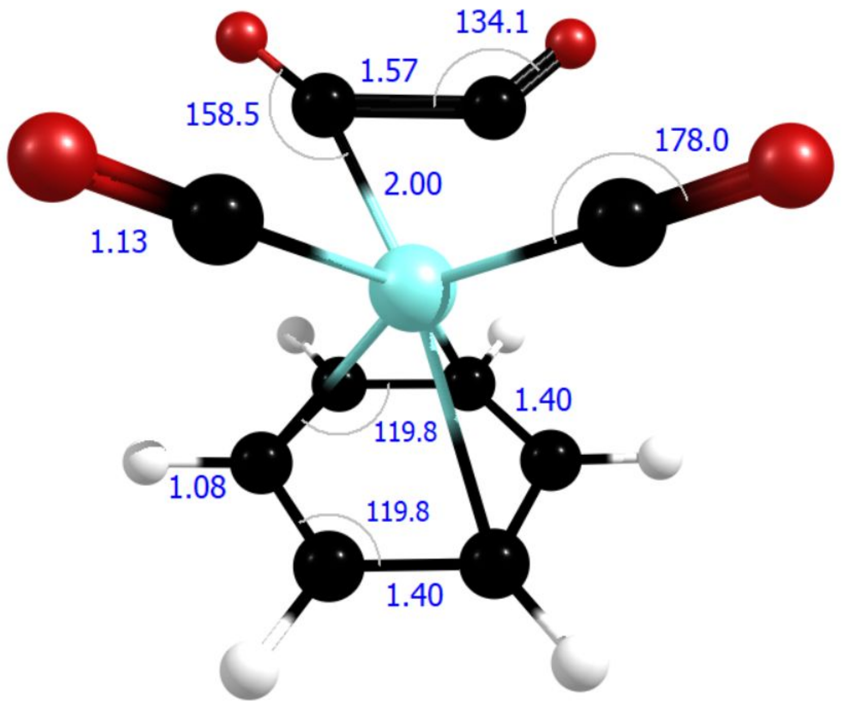

Figure S15. The optimized geometry of isomer 4C-triplet bz-$V^+(CO)_4$ followed by its predicted frequencies (cm^{-1}) and IR intensities (km/mol).

Frequency	Intensity	Frequency	Intensity	Frequency	Intensity
17.1964	0.2089	441.4738	0.5695	1193.2359	1.6144
30.6061	0.1706	480.689	18.3244	1195.8121	3.5137
69.7193	1.1634	489.5657	34.0627	1196.1186	0.0075
78.3097	0.6045	541.1977	18.9945	1355.2457	2.11
79.196	3.2755	561.8288	0.0299	1386.5568	0.0017
88.4326	0.5941	600.3819	20.3967	1498.1444	21.2492
106.2755	2.4121	611.0016	5.0123	1503.7729	22.2073
202.6113	5.5788	616.7627	0.0054	1573.7636	0.1696
222.9762	1.996	686.5236	0.0455	1586.604	15.6793
226.4733	0.6488	790.1454	54.1998	1834.0122	769.6483
262.5	0.4602	932.9349	0.6525	1845.2162	291.0137
280.442	4.6244	935.5795	2.5613	2158.0932	467.439
324.4928	1.2535	993.7514	0.4529	2176.1866	607.2498
335.1782	0.0531	1023.2599	0.0315	3197.4439	0.2174
379.9392	3.7367	1023.7633	0.0498	3201.3422	0.2779
388.4214	6.3959	1025.3131	0.0283	3204.6336	0.2037
402.6782	3.9292	1040.234	0.9485	3211.1162	13.4091
418.6217	0.0737	1052.0844	0.058	3213.7849	12.13
432.8913	14.4515	1052.3635	0.0324	3218.021	2.4864

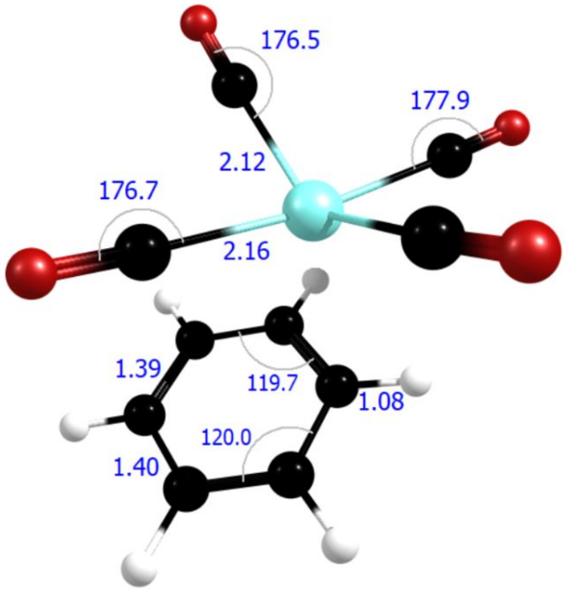

Figure S16. The optimized geometry of isomer 4C-quintet bz-$V^+(CO)_4$ followed by its predicted frequencies (cm^{-1}) and IR intensities (km/mol).

Frequency	Intensity	Frequency	Intensity	Frequency	Intensity
26.2395	0.001	373.7091	0.0055	1181.6997	0.1494
28.6171	0.0111	388.7213	16.361	1201.2158	0.4546
40.3088	0.0681	396.7138	10.2393	1204.1672	0.5271
59.1799	0.1142	403.0309	19.1145	1342.0455	4.111
65.8526	0.3084	416.2442	0.3686	1389.2595	0.1164
68.3134	0.0031	419.5053	0.0023	1507.7445	17.432
71.1797	0.4252	617.3274	0.043	1509.5773	10.9583
72.6295	1.0065	618.3658	0.1471	1611.4318	1.6511
74.0741	0.2684	712.6565	0.0009	1620.3263	1.8992
90.8479	1.2157	726.1123	110.9633	2149.6553	1062.8487
164.055	6.5388	883.0867	0.8785	2165.4919	181.6044
256.3728	0.1683	890.0268	1.6736	2193.7815	857.7404
260.9953	0.0002	978.995	0.0286	2224.0472	92.0975
281.358	0.8941	995.1584	4.9708	3161.4967	0.3489
284.3155	0.3656	1010.9168	0.3169	3174.4732	2.6986
288.7272	0.5595	1029.4174	0.0978	3185.8254	0.0827
302.8033	0.092	1041.1763	0.1301	3192.8103	0.2285
308.9023	13.9031	1051.2074	2.1935	3199.5833	0.2304
319.0483	8.4829	1060.4921	1.0142	3206.809	0.0095

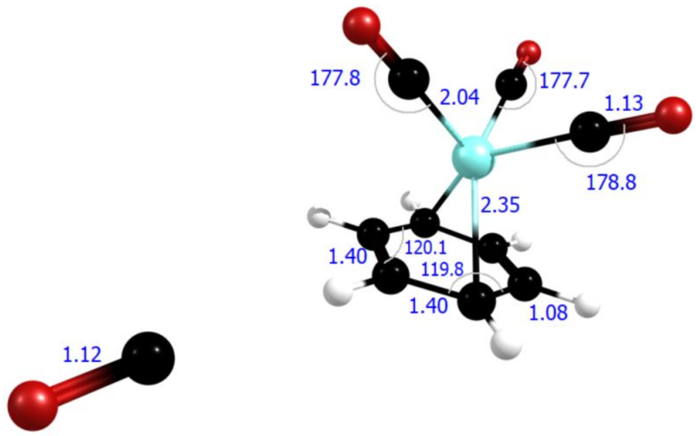

Figure S18. The optimized geometry of isomer (3C + 1)-singlet bz-$V^+(CO)_4$ followed by its predicted frequencies (cm^{-1}) and IR intensities (km/mol).

Frequency	Intensity	Frequency	Intensity	Frequency	Intensity
5.7898	0.3319	395.0036	9.4305	1181.1756	2.3117
11.9266	0.468	417.3049	23.8797	1191.5426	2.9223
17.5597	0.1079	428.3967	27.7773	1196.6792	1.2978
49.4356	2.4785	434.3607	14.6625	1359.2372	11.2163
59.5427	0.1797	502.4924	34.3143	1384.0904	0.0704
71.3115	1.2162	508.2838	21.5781	1492.102	11.2169
77.646	1.9689	607.6231	0.5989	1499.7864	16.3041
80.4893	0.8464	615.2673	0.1141	1548.7992	20.8599
84.0638	0.0136	678.8176	1.1816	1576.8662	21.3571
90.0437	0.2848	795.2161	41.4209	2106.4247	635.1038
96.3669	0.2842	914.9024	0.2884	2114.1549	1087.3014
236.4919	1.8893	928.6077	0.6556	2164.6068	663.5644
247.0901	2.3641	980.2917	7.3552	2241.6065	76.0842
262.9537	3.1537	988.3493	0.7479	3188.9871	31.0428
318.5289	0.3354	1019.3098	0.6108	3199.6768	0.7527
334.3226	9.1786	1028.086	1.6873	3203.9857	6.1642
347.0809	10.6548	1031.3857	4.2819	3210.3181	9.9113
366.8087	10.5722	1049.4214	0.1298	3215.2696	7.7559
388.7648	2.8804	1052.0189	0.2127	3219.3878	3.1432

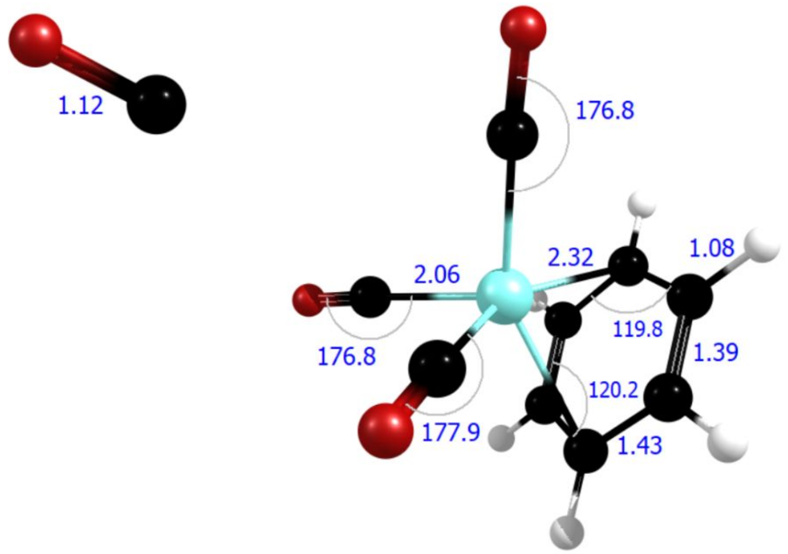

Figure S19. The optimized geometry of isomer (3C + 1)-triplet bz-$V^+(CO)_4$ followed by its predicted frequencies (cm^{-1}) and IR intensities (km/mol).

Frequency	Intensity	Frequency	Intensity	Frequency	Intensity
9.0611	0.2816	384.9039	11.4183	1181.1047	4.7756
13.4045	0.0671	407.211	18.0328	1181.8268	5.0855
18.6231	0.0364	432.3877	34.9453	1188.8219	1.4191
32.6212	1.1739	434.2176	6.5604	1363.6873	7.9933
50.9479	3.6982	487.9558	52.4121	1378.9128	0.0085
63.8047	0.0331	495.8204	16.2277	1487.3968	11.21
71.9134	0.0724	607.6262	0.6931	1492.755	6.9641
73.7218	0.013	618.0369	0.0644	1551.96	24.5836
78.2037	1.087	674.89	0.9968	1555.1985	41.0605
82.8649	1.4072	788.4022	47.8005	2120.7489	631.5083
96.6021	0.4158	905.4338	0.1444	2126.1871	1002.5577
234.5419	7.9086	914.6774	0.9456	2176.917	649.3984
235.6456	1.6633	963.1214	14.1306	2239.2541	78.1034
254.0006	3.3918	984.9147	1.4513	3198.5148	0.5494
313.7871	2.0324	1017.724	0.0344	3200.8636	0.1734
319.1118	2.2153	1017.8282	4.2796	3207.7631	3.7214
335.6349	10.6478	1023.0705	3.2039	3214.056	6.6132
360.6572	2.6209	1043.6968	0.1753	3217.6628	8.8285
369.1935	11.3749	1048.2225	1.4575	3221.7609	2.9742

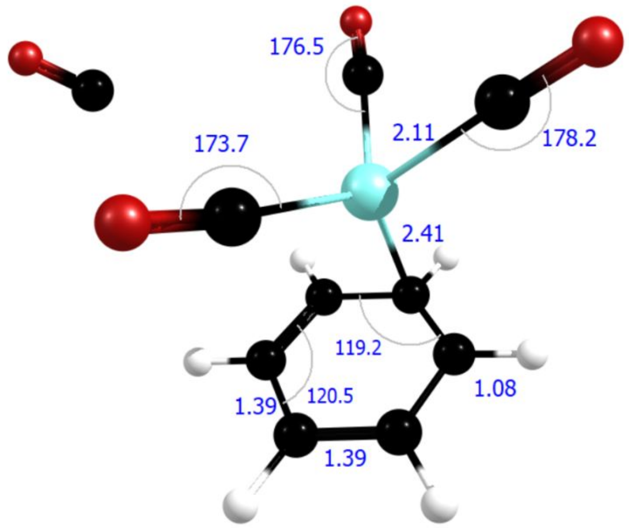

Figure S20. The optimized geometry of isomer (3C + 1)-quintet bz-$V^+(CO)_4$ followed by its predicted frequencies (cm^{-1}) and IR intensities (km/mol).

Frequency	Intensity	Frequency	Intensity	Frequency	Intensity
8.0138	0.1659	316.8807	9.5068	1183.956	0.1832
13.5939	0.2114	338.1201	13.711	1195.9818	0.3016
19.2016	0.3563	368.7593	8.4123	1202.1251	2.1952
25.5303	0.6589	392.5252	8.1514	1326.7232	1.5211
30.0097	0.2886	397.4182	0.6849	1389.2674	0.0248
54.6995	0.3174	428.6201	0.0885	1504.4177	18.8028
58.7916	1.0216	613.0418	0.0152	1509.3932	18.214
62.4555	0.0712	613.944	0.2492	1602.3549	5.435
62.6421	0.0576	701.0718	7.6668	1603.5462	6.0364
65.8987	0.5455	741.0644	91.1773	2147.3145	908.4378
69.3186	0.1937	896.1919	0.2026	2155.3086	542.391
85.7398	1.3266	906.4231	0.2471	2194.8689	432.7532
108.77	0.4333	989.2003	0.665	2238.9796	78.2474
191.9264	3.1235	1004.2244	4.3049	3181.9819	0.2467
260.9235	0.5514	1014.8741	0.9667	3189.3107	0.3144
269.6544	3.2072	1027.96	0.5442	3191.6149	1.0964
286.3276	2.4837	1040.033	1.7439	3200.9454	1.1917
298.9445	0.7782	1049.0121	1.348	3201.4375	0.6365
306.0821	4.8589	1055.0467	1.3166	3210.828	0.2556

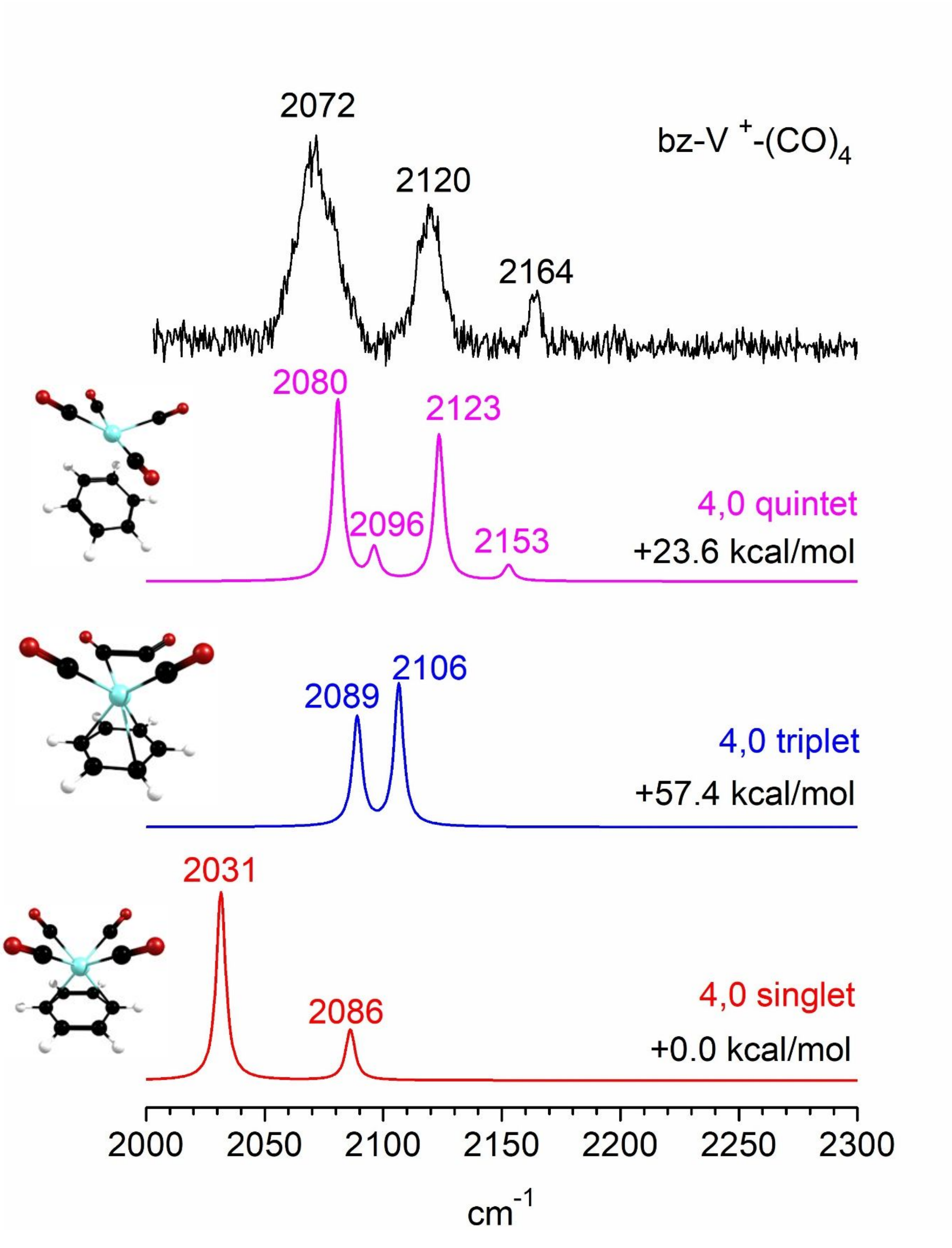

Figure S21. Experimental IR spectrum of bz-V^{+}-$(CO)_4$ compared with simulated spectra for isomer 4C singlet, triplet and quintet. Relative energies (kcal/mol) are shown next to each spectrum.

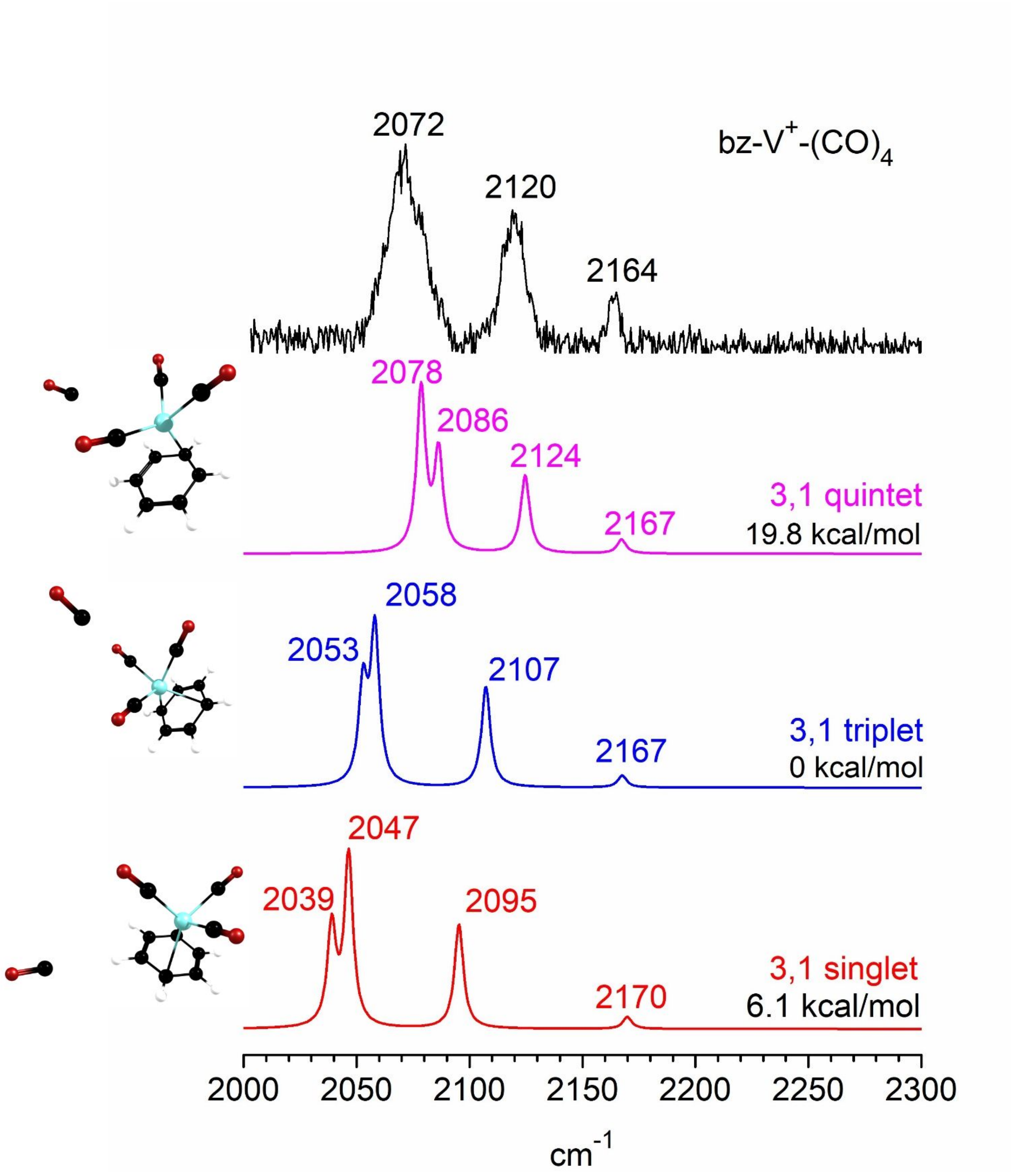

Figure S22. Experimental IR spectrum of bz-V^{+}-$(CO)_4$ compared with simulated spectra for isomer 3C + 1 singlet, triplet and quintet. Relative energies (kcal/mol) are shown next to each spectrum.

Table S7. bz-$V^+(CO)_5$ electronic energy calculated at the B3LYP/def2-TZVP level.

Isomer	2s+1	Energy (Hartree)	Relative Energy (kcal/mol)
4C + 1	1	-1742.919247	0.0
4C + 1	3	-1742.892855	+16.6
4C + 1	5	-1742.881862	+23.5
3C + 2	1	-1742.886847	+6.4
3C + 2	3	-1742.896984	0.0
3C + 2	5	-1742.864893	+20.1

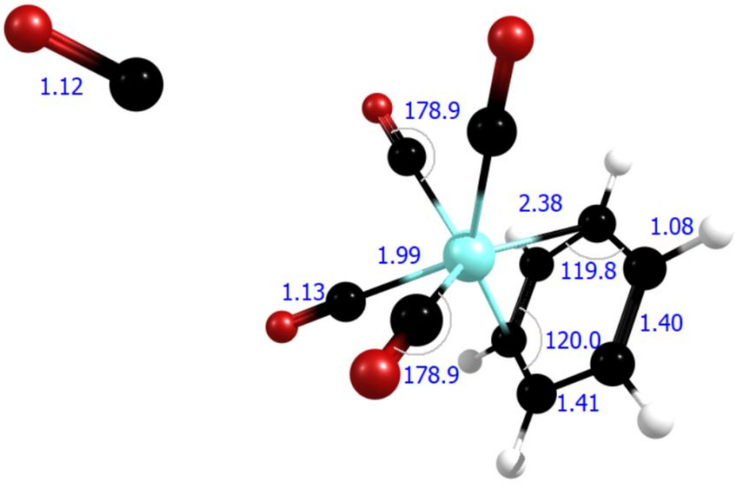

Figure S23. The optimized geometry of isomer (4C + 1)-singlet bz-$V^{+}(CO)_5$ followed by its predicted frequencies (cm^{-1}) and IR intensities (km/mol).

Frequency	Intensity	Frequency	Intensity	Frequency	Intensity
3.8383	0.0003	426.5086	0.8763	1055.7089	0.0132
4.395	0.286	437.3096	0	1191.4526	0.0066
4.5968	0.2857	446.5135	0.0006	1195.246	0.2899
29.1052	0.6573	464.7938	35.6237	1199.223	4.2619
61.9075	0.0301	467.944	34.256	1369.5032	2.4842
61.9274	0.0307	584.0787	49.7061	1387.0771	0
69.8529	0	585.8959	51.0102	1497.9733	21.6329
94.3031	0.918	587.1914	0.0116	1507.5174	23.2907
94.6988	0.9181	610.3913	4.0349	1569.7829	0.0348
104.0586	3.8629	615.741	0.2512	1595.3656	21.601
109.5178	0.0107	616.9466	52.9752	2096.8605	1073.7232
118.8856	0.6633	688.7453	0.0279	2097.3421	1069.0096
120.4446	0.4344	800.79	40.3772	2109.7269	0.0011
237.357	0.4966	931.7958	2.4404	2154.5151	620.1885
245.183	2.1211	943.7911	2.9097	2235.5727	83.2129
269.0536	0.2194	996.7328	0.1607	3197.94	0.4307
327.5844	0	1009.7415	4.7218	3201.172	0.0008
368.6908	5.8796	1025.2116	0.0366	3205.5273	0.2141
376.633	0.0011	1033.1693	0.0118	3211.1512	7.8781
408.6333	7.6475	1037.5228	0.3488	3215.7984	8.7768
409.2484	9.8297	1054.3558	0.085	3219.0176	0.9785

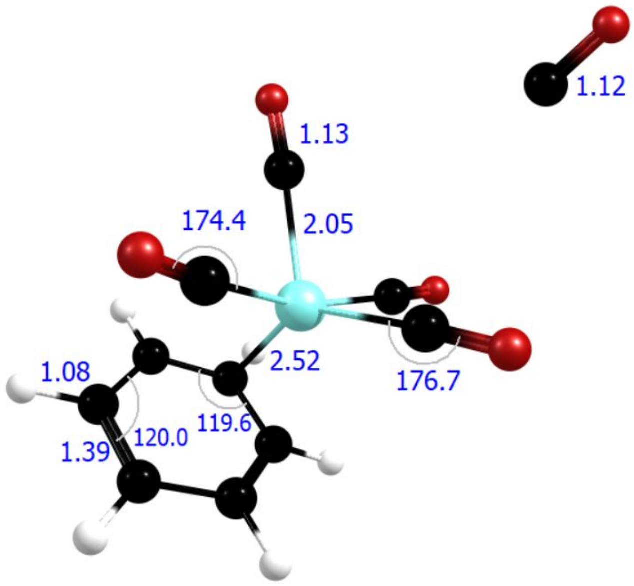

Figure S24. The optimized geometry of isomer (4C + 1)-triplet bz-$V^{+}(CO)_5$ followed by its predicted frequencies (cm^{-1}) and IR intensities (km/mol).

Frequency	Intensity	Frequency	Intensity	Frequency	Intensity
11.2511	0.0093	361.4284	29.3621	1053.3838	1.0837
17.3686	0.0019	373.0066	28.4247	1189.2532	0.7415
32.2679	0.8077	379.8163	7.2478	1201.7814	0.0003
32.4688	0.0317	413.3046	0.2768	1202.0855	0.2448
43.6227	0.276	420.4329	0.1081	1343.968	2.3522
53.0126	0.3213	467.4645	11.9074	1390.7586	0.0243
72.1283	0.1162	486.887	0.0601	1505.3986	22.8055
72.6685	0.3803	496.003	25.7215	1508.5626	15.7168
73.7416	0.0881	536.7522	50.1537	1600.5344	0.5474
77.3118	0.0663	613.5993	0.0678	1612.8049	2.7359
83.889	1.4481	614.0809	0.0611	2117.5629	1234.341
86.7335	2.3412	700.8112	0.006	2124.2399	966.0995
90.3987	0.9036	747.5103	98.8833	2135.2637	84.8588
93.4967	0.1044	907.5046	2.147	2188.5681	379.998
102.73	0.7412	908.4793	0.4935	2239.4808	79.6579
184.713	6.0694	993.9026	5.3818	3186.322	0.0398
304.085	0.0978	1003.7126	0.3141	3192.3804	0.5505
308.2548	1.8518	1022.8914	0.2301	3194.5366	0.0366
322.516	0.5375	1030.7993	0.2232	3203.7693	1.6062
333.2417	0.0353	1041.8633	0.2305	3204.9521	3.0221
339.3481	6.9977	1053.1754	0.9224	3211.2324	0.2855

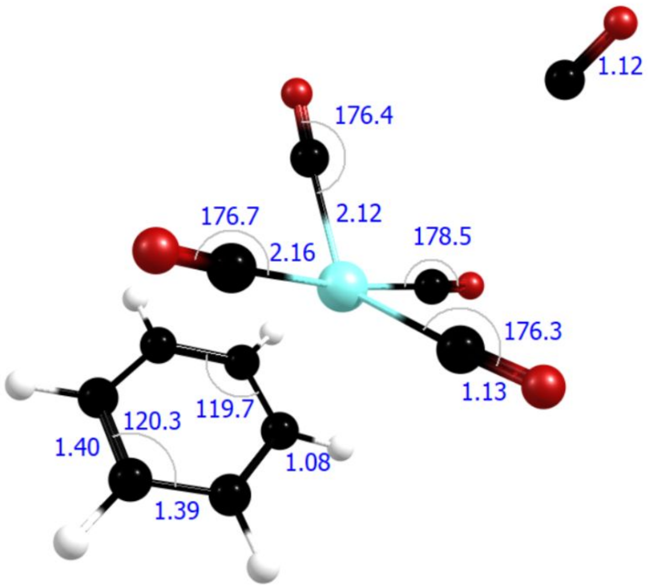

Figure S25. The optimized geometry of isomer (4C + 1)-quintet bz-$V^+(CO)_5$ followed by its predicted frequencies (cm^{-1}) and IR intensities (km/mol).

Frequency	Intensity	Frequency	Intensity	Frequency	Intensity
4.7592	0.0737	305.242	0.1587	1060.6492	1.0337
13.4336	0.0882	309.1919	12.9595	1181.7267	0.1794
26.1759	0.0788	319.9886	7.8575	1201.0939	0.4589
28.4316	0.4364	373.2561	0.0381	1204.1753	0.5237
34.6946	0.4659	388.0965	16.3423	1342.002	4.1766
40.1719	0.0874	396.0005	12.6884	1389.2481	0.1134
61.0276	0.0439	403.0301	19.0623	1507.8119	17.2179
64.3699	0.0424	416.3535	0.3841	1509.5734	10.908
66.264	0.2467	419.5959	0.004	1611.4788	1.7197
67.6744	0.0384	617.4053	0.0504	1620.5661	1.9153
68.242	0.0132	618.4075	0.1476	2147.2466	1038.423
71.9207	0.8444	712.6186	0.0022	2164.5181	196.9933
73.4903	0.5928	725.4502	111.4846	2194.3425	849.4805
74.2465	0.2589	882.5582	0.8825	2224.1976	91.9394
91.2054	1.5734	889.1492	1.6313	2238.2393	79.3299
162.7567	6.6204	978.6575	0.0332	3161.7471	0.3428
256.4155	0.145	995.0668	4.818	3174.7206	2.9658
261.1983	0.0009	1010.4657	0.3413	3185.7044	0.0824
280.1313	0.891	1029.2126	0.0922	3192.7912	0.2795
285.608	0.3264	1040.6645	0.1305	3199.5565	0.2874
288.8143	0.4017	1051.1346	2.226	3206.7531	0.0171

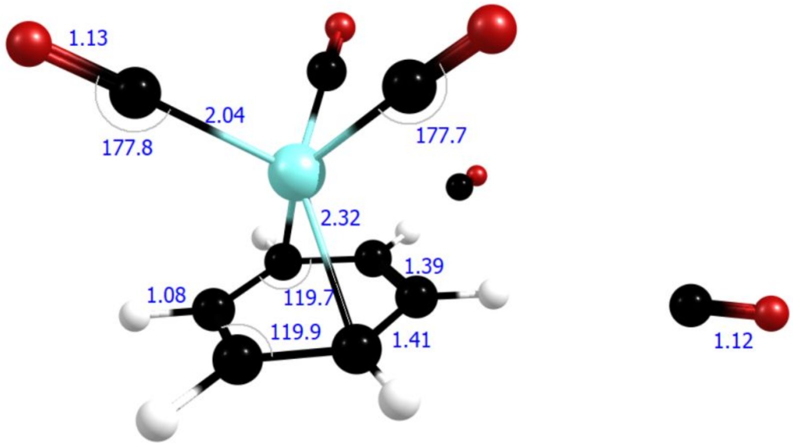

Figure S26. The optimized geometry of isomer (3C + 2)-singlet bz-$V^+(CO)_5$ followed by its predicted frequencies (cm^{-1}) and IR intensities (km/mol).

Frequency	Intensity	Frequency	Intensity	Frequency	Intensity
6.5239	0.0486	348.032	10.3371	1054.1949	0.6404
7.482	0.1648	367.7318	10.1207	1181.5344	2.9303
10.4674	0.3833	389.3276	2.6052	1188.8217	0.7357
13.2752	0.4387	398.2651	9.3503	1204.966	4.3997
22.2567	0.0236	415.7427	21.8009	1359.1759	10.8101
46.1581	1.3592	429.7869	31.1369	1386.4005	0.0285
49.5838	3.0213	434.8731	14.6312	1494.335	13.5799
60.04	0.4094	502.9985	33.0574	1500.8576	14.8968
71.4854	1.1404	509.3282	22.4058	1546.6896	13.7561
77.605	1.8086	607.674	0.2903	1580.1664	23.9776
79.5181	0.8072	615.4528	1.0272	2104.4757	640.3395
81.2767	0.0841	679.5578	1.336	2111.8787	1092.3181
82.0723	0.0262	799.3036	38.133	2162.9148	666.7552
84.2193	0.0604	916.0987	0.4322	2240.1589	50.7825
89.6526	0.412	939.0189	0.2588	2240.8083	102.6413
100.1339	0.0916	988.0277	2.8442	3190.17	21.175
236.9393	1.7526	989.1088	5.9859	3197.8648	0.6692
246.6636	2.5817	1017.354	3.0687	3198.8154	34.6241
266.8044	2.9646	1029.2734	1.5412	3206.2828	18.4847
319.9152	0.53	1034.1085	0.5239	3214.1674	8.352
336.5741	9.8966	1051.4803	0.1705	3218.726	3.8473

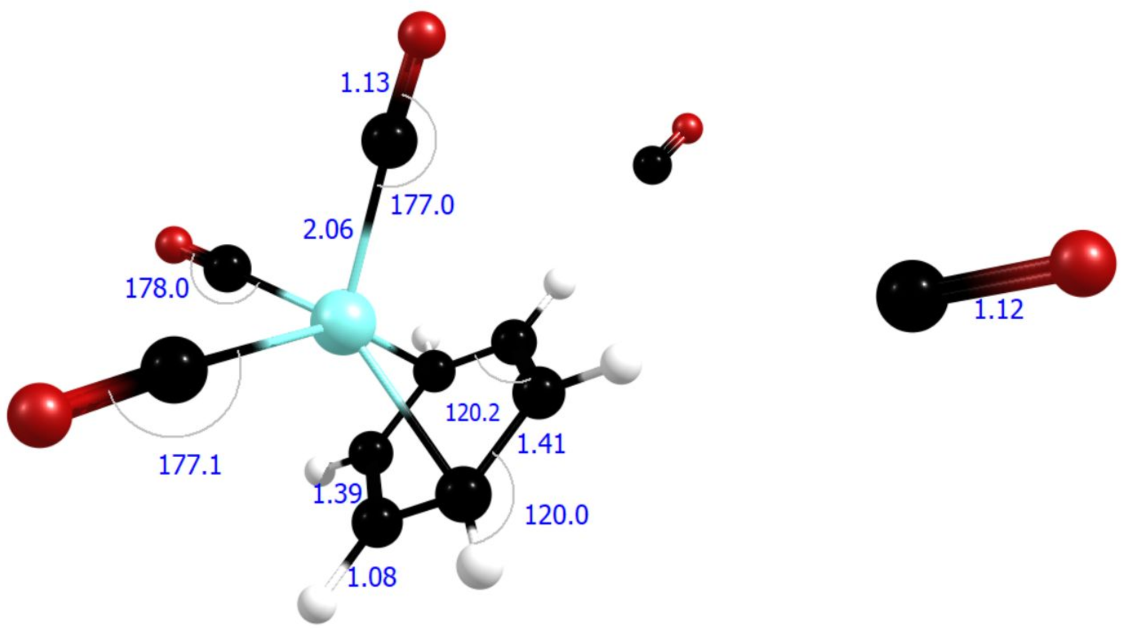

Figure S27. The optimized geometry of isomer (3C + 2)-triplet bz-$V^{+}(CO)_5$ followed by its predicted frequencies (cm^{-1}) and IR intensities (km/mol).

Frequency	Intensity	Frequency	Intensity	Frequency	Intensity
6.9111	0.1859	337.7284	10.5004	1051.1836	0.4495
7.6146	0.1577	361.7118	3.5258	1179.7431	5.3089
10.3815	0.3833	370.428	14.1258	1188.8284	3.9068
13.3446	0.5592	386.9024	11.3751	1198.444	1.2376
23.374	0.0305	409.6659	17.9913	1362.9215	8.4958
45.2898	1.1141	432.0633	17.007	1384.4656	0.1122
47.6685	3.5182	435.7047	22.795	1490.8479	9.3639
48.8514	2.6427	487.9136	40.0167	1496.7229	10.8398
63.1967	0.1727	495.5773	28.0881	1543.6362	33.5859
76.9321	0.6708	608.4619	1.0745	1568.2067	29.7922
79.8308	0.6976	617.5684	0.2711	2116.515	699.9784
80.4338	0.4954	676.9173	1.017	2121.5053	1069.4134
82.0463	0.0491	797.2695	41.3559	2173.5261	620.0711
82.8436	0.3187	911.0793	0.0627	2239.8392	55.9084
87.1735	0.2876	926.7689	2.1731	2240.6339	98.1556
99.5745	0.2038	978.6907	11.6885	3188.8235	18.3387
235.7369	2.2371	985.5451	0.4886	3198.5329	31.3725
238.961	5.322	1016.8097	1.3013	3200.5261	17.4807
258.0027	3.8812	1027.0056	2.6022	3210.3746	9.0451
314.2657	2.4397	1032.1307	4.2357	3215.2783	10.7265
323.2858	2.372	1047.5528	0.7039	3220.1659	3.5297

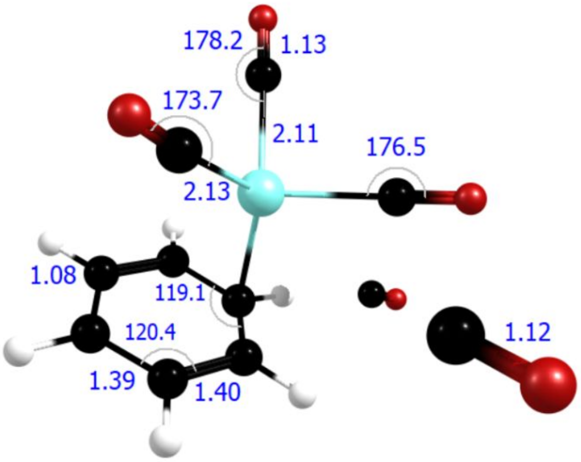

Figure S28. The optimized geometry of isomer (3C + 2)-quintet bz-$V^{+}(CO)_5$ followed by its predicted frequencies (cm^{-1}) and IR intensities (km/mol).

Frequency	Intensity	Frequency	Intensity	Frequency	Intensity
5.6907	0.2544	286.9714	2.4989	1055.3363	0.9778
9.2986	0.1814	299.6829	0.8115	1187.1004	0.6052
10.7634	0.2693	306.594	4.9085	1198.973	0.0533
14.5895	0.1489	317.5232	9.5605	1201.9146	2.0652
22.0664	0.579	339.0389	13.5256	1326.9297	1.5004
26.3936	0.3769	370.0137	8.847	1391.9263	0.0025
29.7752	0.2588	393.0229	8.1598	1507.3533	17.5844
41.5512	1.1146	397.6212	0.6567	1509.3728	20.0114
56.2107	0.8435	430.9902	0.1038	1602.5453	5.8421
58.9286	0.9746	613.4659	0.2877	1603.8483	5.3519
61.7914	0.0379	613.8223	0.0306	2145.7024	931.9725
61.9719	0.0647	702.1443	6.6667	2153.4743	539.7334
65.9172	0.5288	744.3171	88.8861	2193.5255	431.8062
68.843	0.1707	905.902	0.2366	2238.4152	91.9521
76.2639	0.0611	907.3852	0.4559	2239.1125	66.9178
77.0771	0.0329	992.3624	0.5959	3179.1255	12.4987
88.5547	1.2772	1011.2218	3.2039	3188.3539	1.336
110.0873	0.4477	1014.7865	0.9062	3189.1303	6.8132
194.9002	3.5225	1027.9103	1.0419	3198.3709	4.8638
261.3218	0.4734	1041.7028	2.1132	3200.7514	0.2899
271.033	3.3105	1051.3278	1.1047	3210.3727	0.1306

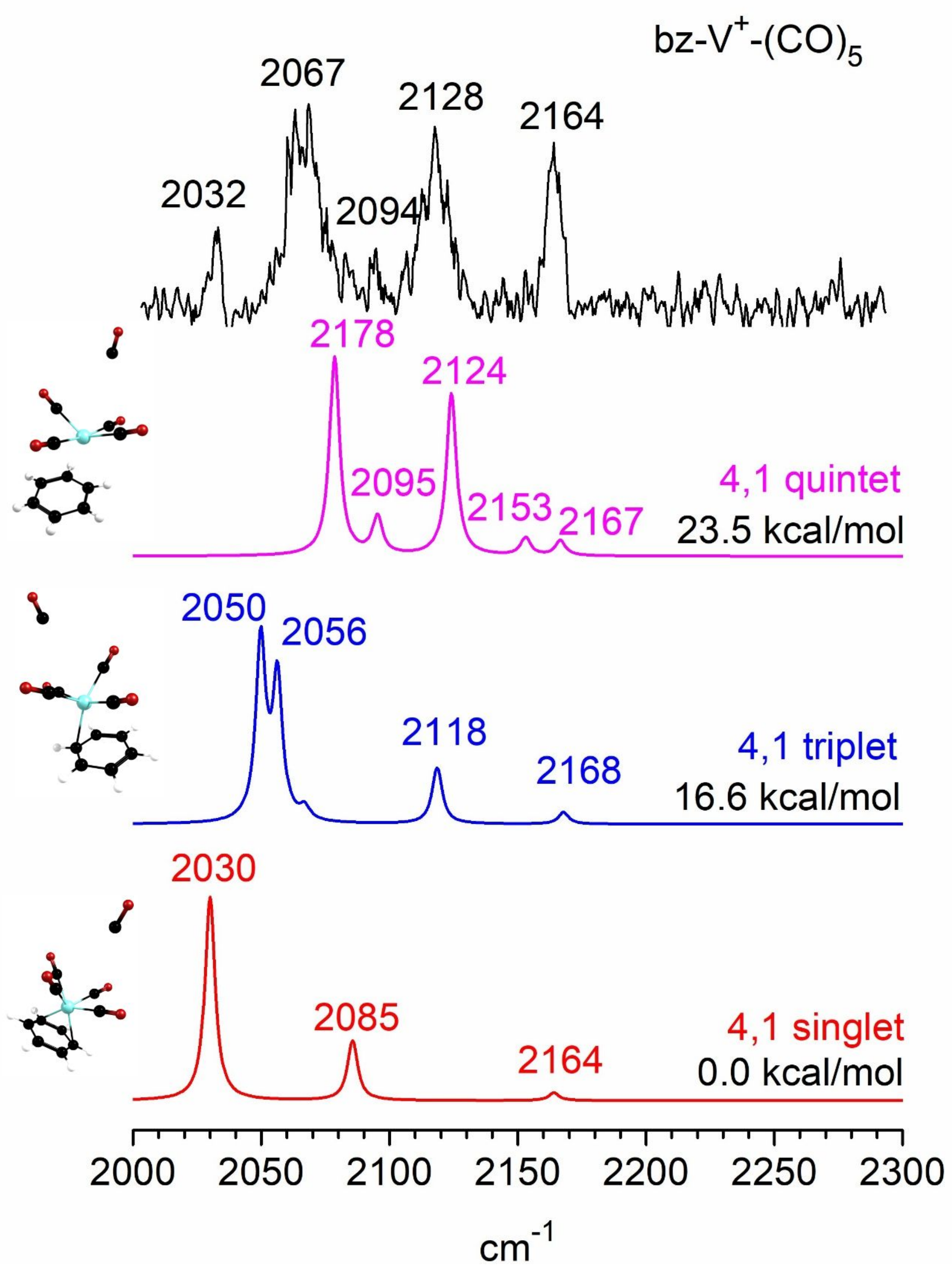

Figure S29. Experimental IR spectrum of bz-V^{+}-$(CO)_5$ compared with simulated spectra for isomer 4C + 1 singlet, triplet and quintet. Relative energies (kcal/mol) are shown next to each spectrum.

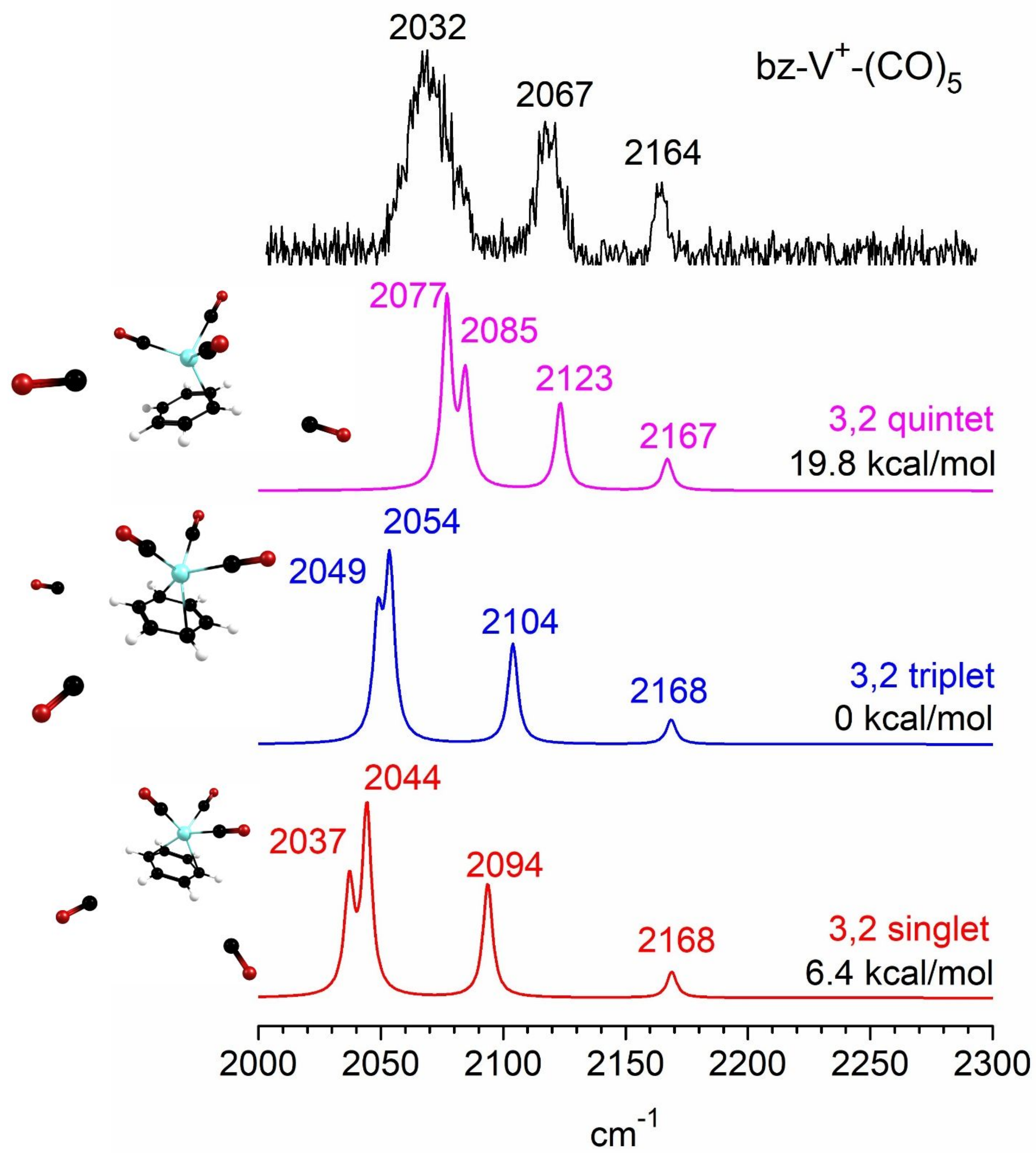

Figure S30. Experimental IR spectrum of bz-V^{+}-$(CO)_5$ compared with simulated spectra for isomer 3C + 2 singlet, triplet and quintet. Relative energies (kcal/mol) are shown next to each spectrum.

Table S8. bz-$V^+(CO)_6$ electronic energy calculated at the B3LYP/def2-TZVP level.

Isomer	2s+1	Energy (Hartree)	Relative Energy (kcal/mol)
3C + 3	1	-1856.245830	+6.4
3C + 3	3	-1856.256043	0.0
3C + 3	5	-1856.223275	+20.6

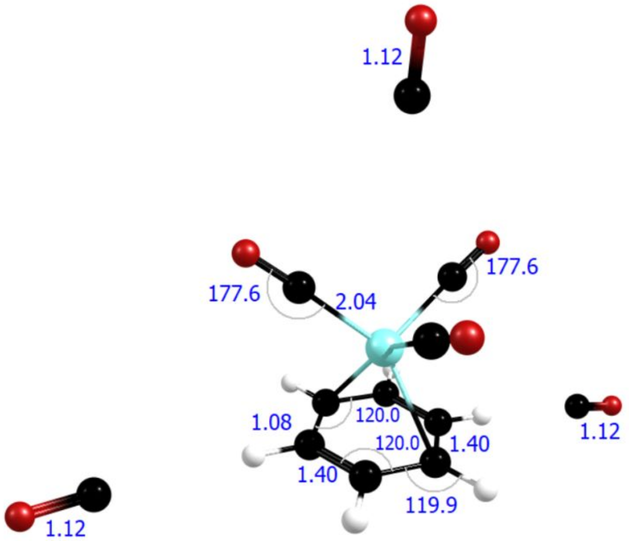

Figure S31. The optimized geometry of isomer (3C + 3)-singlet bz-$V^+(CO)_6$ followed by its predicted frequencies (cm^{-1}) and IR intensities (km/mol).

Frequency	Intensity	Frequency	Intensity	Frequency	Intensity
4.2724	0.0993	261.0711	2.9066	1049.9004	0.0073
7.3064	0.8248	318.3703	0.1491	1052.6894	0.2261
8.7677	0.3088	333.7543	8.2485	1184.0886	3.1158
9.5515	0.2502	347.6186	9.1476	1195.8394	2.3549
10.8601	0.3099	367.9524	10.0138	1196.1705	0.9571
18.4111	0.0978	388.7318	3.2513	1359.4122	11.5462
21.4169	0.0218	393.6245	8.8559	1386.7399	0.0359
30.39	0.9891	422.0884	27.1824	1497.3275	9.2642
44.4796	0.1417	426.566	27.5389	1497.3666	18.2581
52.1399	4.5656	436.0214	13.9401	1555.1372	23.0292
60.3945	0.2292	502.9556	40.7518	1570.511	22.2036
65.7407	0.2872	509.6785	17.7936	2103.6987	612.6738
67.3036	0.0166	608.5008	0.6863	2112.1817	1061.2782
72.5303	1.3981	615.2715	0.2254	2163.5178	693.3127
78.3498	1.8789	679.6324	1.3661	2236.4154	82.1862
78.9114	0.2548	798.4473	37.8571	2240.6145	151.7043
82.3413	0.0218	915.2345	0.4115	2240.8684	2.8443
83.1782	0.0001	933.882	0.5289	3189.6575	56.0058
86.218	0.1094	976.6323	8.1058	3190.8612	1.8245
92.2056	0.1668	988.1233	0.6529	3203.8224	11.1931
96.5016	0.313	1027.1471	1.721	3209.1769	13.7864
237.7028	1.4814	1029.5167	1.3945	3213.758	6.3196
250.1388	2.5157	1036.4781	3.5322	3218.0241	3.4481

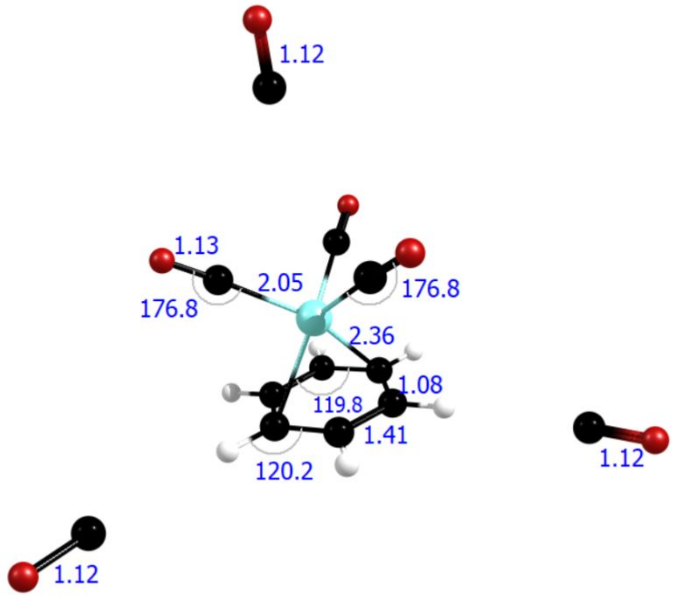

Figure S32. The optimized geometry of isomer (3C + 3)-triplet bz-$V^{+}(CO)_6$ followed by its predicted frequencies (cm^{-1}) and IR intensities (km/mol).

Frequency	Intensity	Frequency	Intensity	Frequency	Intensity
5.4129	0.3046	255.9016	3.467	1047.0079	0.3305
6.7614	0.4452	315.0599	1.9211	1050.4213	0.443
8.8466	0.2916	322.4849	2.8564	1184.4218	6.2255
9.5781	0.3775	337.4446	9.3063	1187.4636	4.2065
14.6138	0.1815	362.3642	2.2274	1194.51	0.7653
19.1566	0.0987	371.6497	12.0483	1363.3795	7.7901
22.3405	0.0544	386.3461	11.5642	1384.6818	0.1931
31.8368	1.0548	408.3686	19.693	1490.6403	10.9069
45.5555	1.1466	434.0504	36.9398	1496.4539	10.0913
49.1727	4.0611	436.7506	4.6489	1552.9116	28.9705
52.048	2.5352	489.7472	52.7964	1558.255	33.9358
63.9429	0.1376	496.5204	16.2147	2116.4294	652.301
70.4871	0.0313	608.6182	1.4323	2121.4892	1047.6835
72.3259	0.0124	617.928	0.4532	2173.6785	640.6795
77.6916	0.4168	676.9638	0.9736	2238.2921	83.0008
80.4536	0.0101	797.1195	41.527	2239.9639	98.6764
81.0187	0.7795	912.2438	0.14	2240.4897	54.2906
82.2649	0.0281	923.0322	2.3391	3189.8649	27.1061
85.0044	0.7066	975.2876	12.1617	3196.2766	24.0413
87.4616	0.2496	985.1854	0.8731	3202.8522	18.4868
99.0516	0.2332	1020.1534	1.0605	3211.7591	8.3679
236.7169	1.401	1025.5364	1.7336	3213.2134	13.897
238.7299	6.8339	1032.7354	3.9061	3220.0295	3.4584

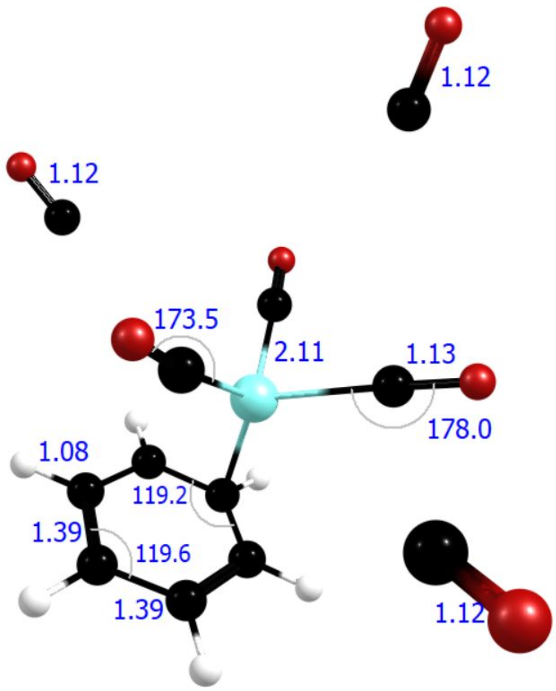

Figure S33. The optimized geometry of isomer (3C + 3)-quintet bz-$V^+(CO)_6$ followed by its predicted frequencies (cm^{-1}) and IR intensities (km/mol).

Frequency	Intensity	Frequency	Intensity	Frequency	Intensity
3.7292	0.2003	191.3558	2.736	1049.0747	1.4612
5.8056	0.1913	260.6735	0.8073	1055.0655	1.3295
6.812	0.1598	268.2217	2.9046	1183.7987	0.2308
12.0752	0.1397	286.4225	2.2948	1195.3658	0.336
15.543	0.1325	298.8106	0.4814	1202.0665	2.47
16.6275	0.2444	307.8043	4.4985	1326.1328	1.7042
20.3553	0.5096	317.7662	8.5672	1389.1454	0.0244
25.3261	0.2372	337.7823	14.44	1504.4308	18.2453
27.7067	0.587	367.4281	9.5902	1509.3648	17.8151
28.0823	0.8532	392.9675	9.0975	1602.3414	4.7373
33.4232	0.6378	396.8064	0.8737	1603.3334	7.6182
55.2444	0.4225	428.6982	0.0752	2144.8045	918.8155
58.1394	0.4137	613.0756	0.0111	2155.4533	492.4186
59.8499	0.1169	614.0065	0.314	2195.6704	456.468
61.376	0.0184	701.442	8.5543	2236.0332	56.887
61.6915	0.1996	739.7603	92.2965	2236.5955	91.7534
63.8242	0.0637	894.4202	0.2848	2237.8159	83.5287
64.4709	0.1536	905.2773	0.1429	3182.482	0.2555
65.7687	0.0368	988.6084	0.8199	3189.8483	0.3259
67.7677	0.385	1004.1985	4.2062	3192.0323	1.0678
71.7519	0.1553	1014.1023	0.9679	3201.442	1.086
86.4055	1.4571	1027.3291	0.656	3202.031	0.3961
109.1614	0.4958	1039.5833	1.8109	3211.1865	0.2

Table S9. bz-$V^+(CO)_7$ electronic energy calculated at the B3LYP/def2-TZVP level.

Isomer	2s+1	Energy (Hartree)	Relative Energy (kcal/mol)
4C + 3	1	-1969.638535	0.0
3C + 4	3	-1969.615358	+14.5
3C + 4	5	-1969.582386	+35.2

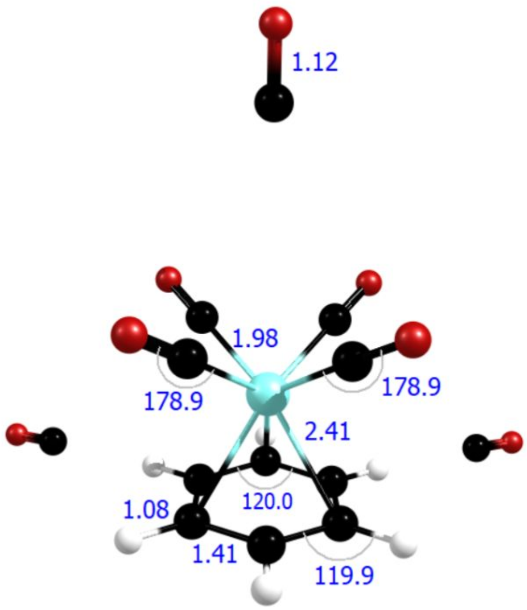

Figure S34. The optimized geometry of isomer (4C + 3)-singlet bz-$V^+(CO)_7$ followed by its predicted frequencies (cm^{-1}) and IR intensities (km/mol).

Frequency	Intensity	Frequency	Intensity	Frequency	Intensity
2.8491	0.0657	272.1513	0.5362	1048.4997	0.0807
4.7668	0.0202	329.1986	0.0073	1056.5403	0.4564
5.0263	0.0431	370.8305	5.1538	1062.6053	0.0625
7.0584	0.4537	378.7837	0.001	1191.8323	0.2284
11.5153	0.7582	410.2361	7.1065	1202.807	0.0091
12.3206	0.5948	410.9387	9.3406	1206.769	4.4297
25.4741	0.0089	428.4719	1.3765	1369.5826	2.5737
27.4734	0.5947	438.9478	0.0038	1393.1578	0.141
48.4458	1.1755	448.6407	0.0066	1503.6158	19.9883
52.2338	3.2575	466.1213	36.1389	1509.0489	27.4175
60.6762	0.0268	469.2438	37.0399	1570.636	0.4177
60.8954	0.0092	585.288	49.459	1596.2314	21.1045
70.2905	0.0001	587.0894	53.027	2093.4747	1114.5914
83.9178	0.0029	588.2357	0.1049	2093.9678	1082.3985
84.813	0.0002	611.67	3.3599	2106.4343	0.3326
87.2568	0.0196	615.5237	0.6885	2152.118	613.9925
88.1721	0.0512	618.3853	52.5144	2234.6205	85.3749
95.507	1.1678	690.263	0.0427	2241.5817	114.8525
96.0229	1.7202	809.6238	34.1999	2241.8534	38.4924
104.6797	3.5311	936.0789	2.2166	3189.2875	18.213
109.5017	0.0157	955.9775	3.1857	3190.7836	48.1295
120.0777	0.4982	996.7446	0.599	3202.2385	0.9177
123.174	0.1477	1014.0564	3.8666	3206.7113	15.4365
239.2202	0.2783	1028.0982	0.6192	3213.0642	9.4766
247.2824	2.1985	1041.5208	0.0827	3216.6283	1.7196

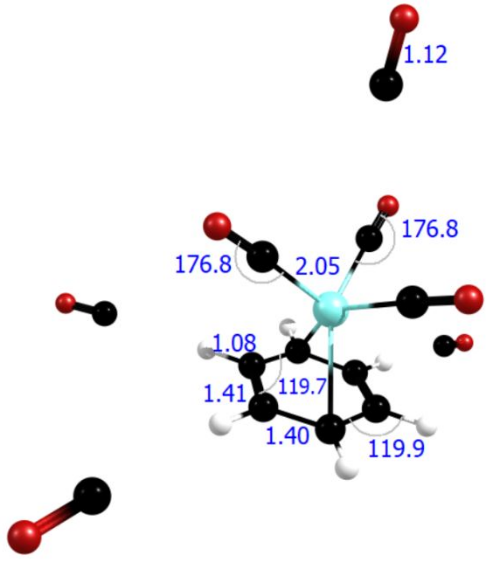

Figure S35. The optimized geometry of isomer (3C + 4)-triplet bz-$V^+(CO)_7$ followed by its predicted frequencies (cm^{-1}) and IR intensities (km/mol).

Frequency	Intensity	Frequency	Intensity	Frequency	Intensity
6.7332	0.3372	99.2662	0.2167	1037.664	3.9163
7.1553	0.0409	237.4313	1.2381	1047.5114	0.2151
7.469	0.1705	241.8946	6.1706	1051.1143	0.3624
8.2609	0.2554	256.7617	3.3193	1184.0587	6.2208
9.1651	0.3977	315.6204	1.8548	1192.0022	3.9994
9.9705	0.3292	324.4069	3.1526	1196.9528	0.0508
14.643	0.1996	338.2541	8.7577	1363.0622	8.012
19.4989	0.0875	363.149	2.1984	1387.1637	0.1778
26.5375	0.0858	372.807	12.3421	1494.7642	10.6747
31.5891	0.9916	387.0338	11.8036	1495.724	10.208
43.1167	0.1777	409.0479	20.6021	1551.3385	27.0077
44.9205	1.357	434.7555	37.396	1561.0519	38.2757
51.209	6.5469	438.6269	4.8234	2114.4112	656.4138
52.9712	1.3384	490.3157	49.7658	2119.4717	1066.5935
64.0271	0.1663	497.338	18.4984	2172.1658	638.2447
69.548	0.0147	609.5113	1.2744	2237.7699	84.9724
71.8041	0.0141	617.4542	0.3364	2238.9009	55.9265
77.0026	0.2454	677.8298	1.1066	2239.6197	162.4297
78.607	0.0352	800.7591	38.0142	2240.0439	13.2342
80.2382	0.4842	912.5329	0.0882	3189.5281	37.0068
80.7317	0.0645	929.9763	2.6489	3191.071	7.2861
81.7162	0.0319	976.4628	11.8777	3200.1127	49.3118
82.103	0.383	985.2841	0.613	3209.7101	10.0494
85.9021	0.4888	1026.3962	1.2101	3213.2255	12.75
89.2554	0.2138	1028.1076	2.1628	3218.9511	3.6714

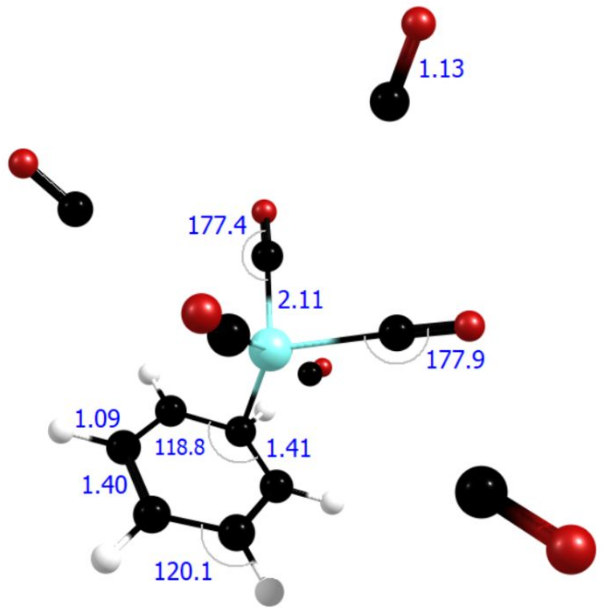

Figure S36. The optimized geometry of isomer (3C + 4)-quintet bz-$V^+(CO)_7$ followed by its predicted frequencies (cm^{-1}) and IR intensities (km/mol).

Frequency	Intensity	Frequency	Intensity	Frequency	Intensity
3.6597	0.1589	76.5296	0.059	1041.4201	2.311
5.0637	0.1184	88.8825	1.4348	1051.3781	1.2119
5.6608	0.2253	110.3045	0.518	1055.3819	1.0052
7.3216	0.3146	194.2226	3.1308	1186.8106	0.683
10.0956	0.2586	261.1315	0.6509	1198.4222	0.0548
13.0084	0.0637	269.5245	3.0744	1201.8424	2.3481
16.4053	0.2286	287.0142	2.3088	1326.3536	1.678
18.4034	0.1972	299.4831	0.5138	1391.8135	0.0018
21.9874	0.4145	308.3094	4.4939	1507.3512	17.0495
25.4582	0.2126	318.3968	8.6189	1509.3484	19.5552
27.7731	0.8707	338.6395	14.227	1602.6718	5.6338
28.0768	0.4194	368.6845	10.0639	1603.5517	6.3843
33.3725	0.5803	393.4022	9.0984	2143.1814	940.3233
41.3935	1.1991	397.0334	0.8528	2153.7164	490.5052
56.1695	0.7495	430.9536	0.0829	2194.4023	456.471
58.178	0.385	613.5252	0.3185	2235.643	56.201
59.4671	0.1556	613.8679	0.0601	2236.1945	97.2487
60.6403	0.0097	702.5811	7.3978	2237.3293	98.0902
61.3767	0.2022	743.0013	90.4343	2238.4108	63.9929
63.2832	0.062	904.2128	0.3963	3179.8874	11.6364
64.0507	0.1703	906.1049	0.232	3188.909	0.6296
65.3769	0.025	992.0529	0.3335	3189.6209	7.3848
67.59	0.3714	1011.0541	3.3772	3198.8899	4.9114
71.0302	0.1685	1013.9639	0.9249	3201.2968	0.1368
75.8715	0.0031	1027.1564	1.1586	3210.688	0.15
